# Exploring the relationship between preferred bubble tube speeds in sensory rooms and physiological–psychological factors: A study on interoceptive sensitivity, subjective time perception, visual discomfort levels, and anxiety levels

**DOI:** 10.12688/f1000research.161090.1

**Published:** 2025-02-14

**Authors:** Anjie Su, Junyi Shen, Shinichi Koyama

**Affiliations:** 1 Doctoral Program in Design, University of Tsukuba, Tsukuba, Ibaraki Prefecture, Japan; 2 Institute of Art and Design, University of Tsukuba, Tsukuba, Ibaraki Prefecture, Japan

**Keywords:** Sensory Hypersensitivity, Sensory Room, Bubble Tubes, Interoceptive Sensitivity, Subjective Time Perception, Visual Discomfort, Anxiety

## Abstract

**Background:**

This study investigated the relationship between preferred bubble speeds in sensory room bubble tubes and physiological–psychological factors, including interoceptive sensitivity, subjective time perception, visual discomfort, and anxiety levels.

**Methods:**

A sample of 50 participants engaged in a controlled experiment in which they used the method of adjustment to determine their preferred speed of a bubble tube simulated by an upward-moving Random Dot Motion (RDM) stimulus. Subjective time perception was evaluated through a time estimation task in which participants estimated a 60-second interval, and interoceptive sensitivity was measured via a heartbeat-tracking task. Participants’ visual discomfort and anxiety levels were assessed using the Visual Discomfort Scale Japanese Version (VDS-J), Trypophobia Questionnaire Japanese Version (TQ-J), and State-Trait Anxiety Inventory (STAI).

**Results:**

The results from the method of adjustment indicated that the preferred speed varied largely, from 1.09 to 13.86 degrees per second. Spearman’s correlation analysis revealed that higher interoceptive awareness correlated with a preference for slower speeds, whereas higher anxiety levels correlated with a preference for faster speeds. In addition, our multiple regression analysis showed that subjective time perception accuracy and visual discomfort levels were significant predictors of the participants’ preferred RDM speeds in the regression model.

**Conclusion:**

The results indicate that interoceptive sensitivity, subjective time, visual discomfort, and anxiety levels play significant roles in determining the preferred speeds for RDM stimulation. Our results highlight the importance of considering individual differences in physiological and psychological states when designing therapeutic sensory environments such as sensory rooms and bubble tubes to enhance well-being and therapeutic outcomes.

## Introduction

Sensory overload is a common experience in individuals with sensory hypersensitivity, in which everyday stimuli such as light ( Lucker, 2013), noise ( Khalfa et al., 2004; Landon et al., 2016), and touch ( Blakemore et al., 2006) become overwhelming. They may find it challenging to navigate routine environments ( Lucker, 2013), thus affecting their ability to function effectively in daily life ( Kim et al., 2000). Researchers and practitioners have developed various methods and tools to address these problems ( Koegel et al., 2004). These approaches aim not only to reduce stress caused by sensory overload ( Mazurek et al., 2013) but also to improve individuals’ quality of life and functional abilities ( Hannant et al., 2016). Studies have shown that sensory hypersensitivity can significantly influence behavior and emotions and is correlated with psychiatric symptoms ( Engel-Yeger et al., 2016).

Designed to provide a controlled environment that aids in self-regulation and relaxation, sensory rooms are typically equipped with assistive technologies and specialized elements. A prime example of such a space is the Snoezelen room, a multi-sensory environment developed in the Netherlands in the 1970s ( Hulsegge & Verheul, 2005). Snoezelen rooms combine sensory experiences to create a soothing atmosphere where users can explore various stimuli at their own pace, thereby promoting relaxation and reducing stress ( Hulsegge & Verheul, 2005). These environments are particularly beneficial in psychiatric inpatient care where they help individuals manage sensory overload and regulate distress. Research has shown that sensory rooms, including Snoezelen rooms, significantly reduce distress and aid emotional regulation, especially in individuals with a history of aggression or anxiety disorders ( Haig & Hallett, 2023; West et al., 2017). They serve as valuable tools for self-management and offer alternatives to more restrictive psychiatric care practices ( Barbic et al., 2019). In forensic mental health settings, these rooms have been found to reduce patient stress, support recovery, and enhance the overall patient experience within a facility ( Wiglesworth & Farnworth, 2016).

Among the various types of sensory equipment used in these rooms, bubble tubes usually serve as a central element, offering visual, tactile, and auditory stimulation. They are tall, clear cylinders filled with water in which bubbles rise continuously owing to an internal air pump, often accompanied by color-changing LED lights. These tubes are designed to create a calming effect while engaging users visually and tactilely, making them beneficial for individuals with sensory processing disorders, including autism spectrum disorders (ASDs). The continuous movement and changing colors of the bubbles facilitate visual tracking, color recognition, and tactile feedback, which can soothe and attract attention ( Fujisawa, 2021). Unwin et al. (2024) investigated how children with autism use different types of sensory equipment in multi-sensory environments and revealed that bubble tubes are among the most popular.

However, the precise mechanisms by which the bubble tubes induce relaxation remain poorly understood. Although their calming effects are well-documented, the specific factors contributing to these outcomes remain uncertain. It is likely that a combination of sensory stimuli, such as the visual rhythm of the bubbles, changing colors, and gentle auditory stimuli, work in concert to engage multiple senses and promote relaxation. Nonetheless, individual responses to bubble tubes can vary significantly, suggesting that personal factors play a crucial role in shaping the overall experience. As a means of moving closer to elucidating these mechanisms, we explored the physiological and psychological factors that contribute to individual differences in how the effects of bubble tubes are experienced.

To comprehensively understand the determinants of the preferred speed for the bubbles in bubble tubes, we investigated several key physiological and psychological factors: interoceptive sensitivity, subjective time perception, visual discomfort, and anxiety. Each of these variables plays a crucial role in shaping the user’s sensory experience and response to stimuli. Because the underlying mechanism may be complex, we began our investigation by examining the correlations between preferred bubble tube speeds and the aforementioned factors. This approach allowed us to determine whether interoceptive sensitivity, perception of the internal clock, visual discomfort levels, and anxiety were at least partially involved in generating comfort and relaxation. By examining these factors and their correlations, we aimed to elucidate the complex interplay between physiological and psychological states and the effectiveness of sensory interventions. If significant correlations exist, this will provide a foundation for further exploration of the causal relationships and mechanisms underlying preferred bubble tube speeds.

Interoceptive sensitivity, defined as the ability to perceive internal bodily sensations, is fundamental in regulating responses to external stimuli ( Garfinkel & Critchley, 2013). Recent advances in this field suggest that individuals with high interoceptive accuracy who can keenly perceive internal states, such as heartbeat, are better equipped to manage their emotional responses to sensory stimuli ( Tanaka et al., 2021). Pollatos et al. (2012) found that interoceptive sensitivity is associated with a heightened awareness of emotional and physiological states, which influences how individuals respond to environmental stimuli.

Our study examined subjective time perception, a critical facet of cognitive processing influenced by attention, emotion, and sensory input. Research indicates that an individual’s perception of time can significantly affect their mental health and behavioral responses ( Yabe & Yamada, 2023). Given that bubble tubes are designed to enhance sensory processing and well-being, and considering the prevalence of various sensory processing disorders among users, it is highly pertinent to explore the relationship between users’ subjective time perception and their preferred bubble tube speeds.

Visual discomfort can also significantly affect the efficacy of bubble tubes. Research has shown that individuals with high levels of visual discomfort often perform visual tasks more slowly, suggesting that discomfort can significantly affect visual processing efficiency ( Conlon & Humphreys, 2001). Researchers have also explored the neural basis of visual discomfort and have found that when processing uncomfortable stimuli, neural responses tend to be stronger and less frequent. This indicates inefficient neural coding, leading to increased sensory sensitivity and discomfort ( Wilkins, 2016). These findings suggest that bubble tube speed settings should consider the potential for visual discomfort and its neural correlation. Therefore, visual discomfort is a crucial factor in the design of sensory rooms because bubble tube speed can either alleviate or exacerbate this discomfort. By understanding the relationship between users’ visual discomfort levels and their preferred bubble tube speeds, we can tailor the sensory environment to minimize sensory fatigue and enhance comfort.

Anxiety level is another important factor that should be considered as this can significantly affect how individuals interact with their environments. High levels of anxiety may heighten sensitivity to sensory stimuli, making the speed of bubble movement particularly crucial. Research indicates a strong link between anxiety and increased sensitivity to sensory stimuli. For instance, anxiety disorders are associated with heightened sensory processing, in which individuals exhibit increased sensitivity to specific environmental cues ( Åhs et al., 2013). Furthermore, sensory processing sensitivity is correlated with higher levels of anxiety, depression, and stress. This relationship suggests that individuals who process sensory information more intensely experience greater psychological distress, particularly when mindfulness and acceptance levels are low ( Bakker & Moulding, 2012). Assessing user anxiety levels can provide valuable insights into how bubble tubes can be optimized to reduce anxiety and provide a calming effect in sensory rooms.

By examining the correlation between users’ preferred bubble tube speeds and physiological–psychological factors, we can develop a comprehensive understanding of how these factors influence the sensory experience. Understanding these relationships will facilitate the development of effective therapeutic strategies. For instance, by tailoring sensory stimuli, such as adjusting the bubble movement speed to users’ specific anxiety levels, the calming effects of sensory rooms can be enhanced. This optimization approach allows for the creation of sensory environments that better meet the needs of individuals with sensory processing disorders, thereby enhancing comfort and engagement.

## Methods

### Participants

The sample size was determined through a priori power analysis using G*Power (version 3.1). Based on the consideration of three key parameters—a large effect size (r = 0.50) following Cohen’s (1988) guidelines to reflect the anticipated substantive and meaningful association between variables, a conventional significance level (α = 0.05) to maintain an acceptable balance between Type I error rates and the ability to detect true effects, and a statistical power (1-β = 0.80) to ensure an 80% likelihood of detecting a true effect if one existed—G*Power recommended a minimum sample size of 42 participants. To account for potential data loss or participant dropout, we recruited 50 participants to enhance the robustness and reliability of the statistical analyses.

We recruited 50 healthy participants (27 females) aged 22 to 35 years (M = 25.94, SD = 2.74) from the University of Tsukuba between December 25, 2023, and March 22, 2024. Prior to participation, all participants provided written informed consent as approved by the Institutional Review Board (IRB) of the Institute of Art and Design, the University of Tsukuba (IRB No. [GEI021-15]). On the day of the experiment, participants were required to abstain from alcohol, caffeine, and cigarettes to ensure their suitability for the study. Participants reported adequate sleep and normal or corrected-to-normal vision.

### Stimuli

**Figure 1.**
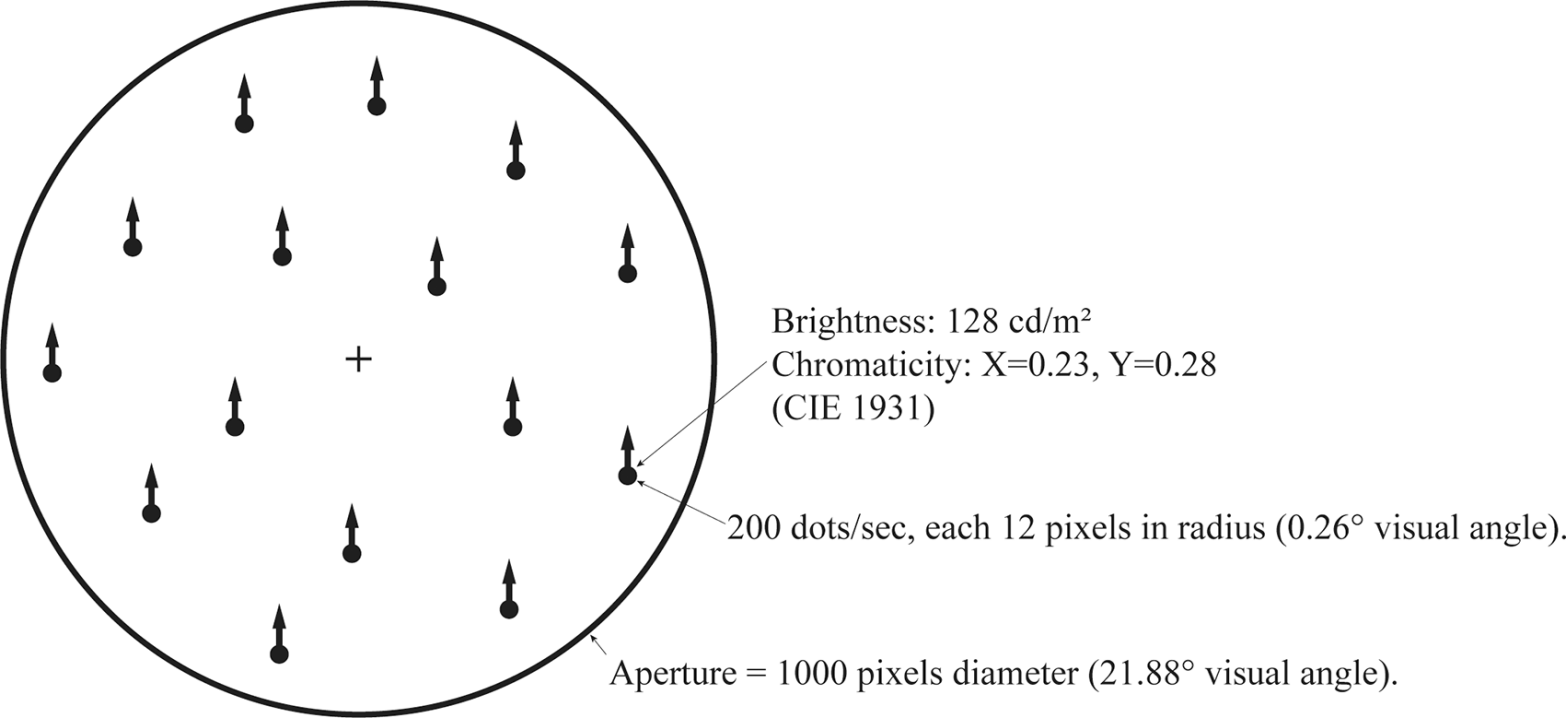
Schematics of the Random Dot Motion (RDM) stimulus.

We used a Random Dot Motion (RDM) stimulus program developed using the Flutter SDK. The source code (v1.0) is available under the MIT License on Zenodo ( Su, 2025c; https://doi.org/10.5281/zenodo.14795461), with a Windows executable build concurrently archived ( Su, 2025d; https://doi.org/10.5281/zenodo.14795194). running on a Lenovo laptop (screen dimensions: 36.3 cm * 23.8 cm, resolution: 1920 * 1080, model number: 115423562). Our RDM program generated a certain number of dots per second that moved vertically upward within a circular area at the center of the screen ( Figure 1). All dots moved at the same speed, simulating the upward movement of the bubbles in the tube. This circular area had a diameter of 1000 pixels, which corresponds to a visual angle of 21.88 degrees. A white fixation cross was placed at the center of the circular area. This helped ensure that participants kept their gazes fixed on a specific location on the screen and their attention focused on the screen, making it easier for them to detect subsequent stimuli. In the experiment, the program was set to randomly generate 200 small dots per second within a circular area. Each dot had a radius of 12 pixels, corresponding to a visual angle of 0.26 degrees. All dots had the same brightness and chromaticity, set at 128 cd/m ^2^, X = 0.23, Y = 0.28 in the CIE 1931 color space, ensuring consistent visual stimuli in the CIE 1931 color space, ensuring consistent visual stimuli.

**Figure 2.**
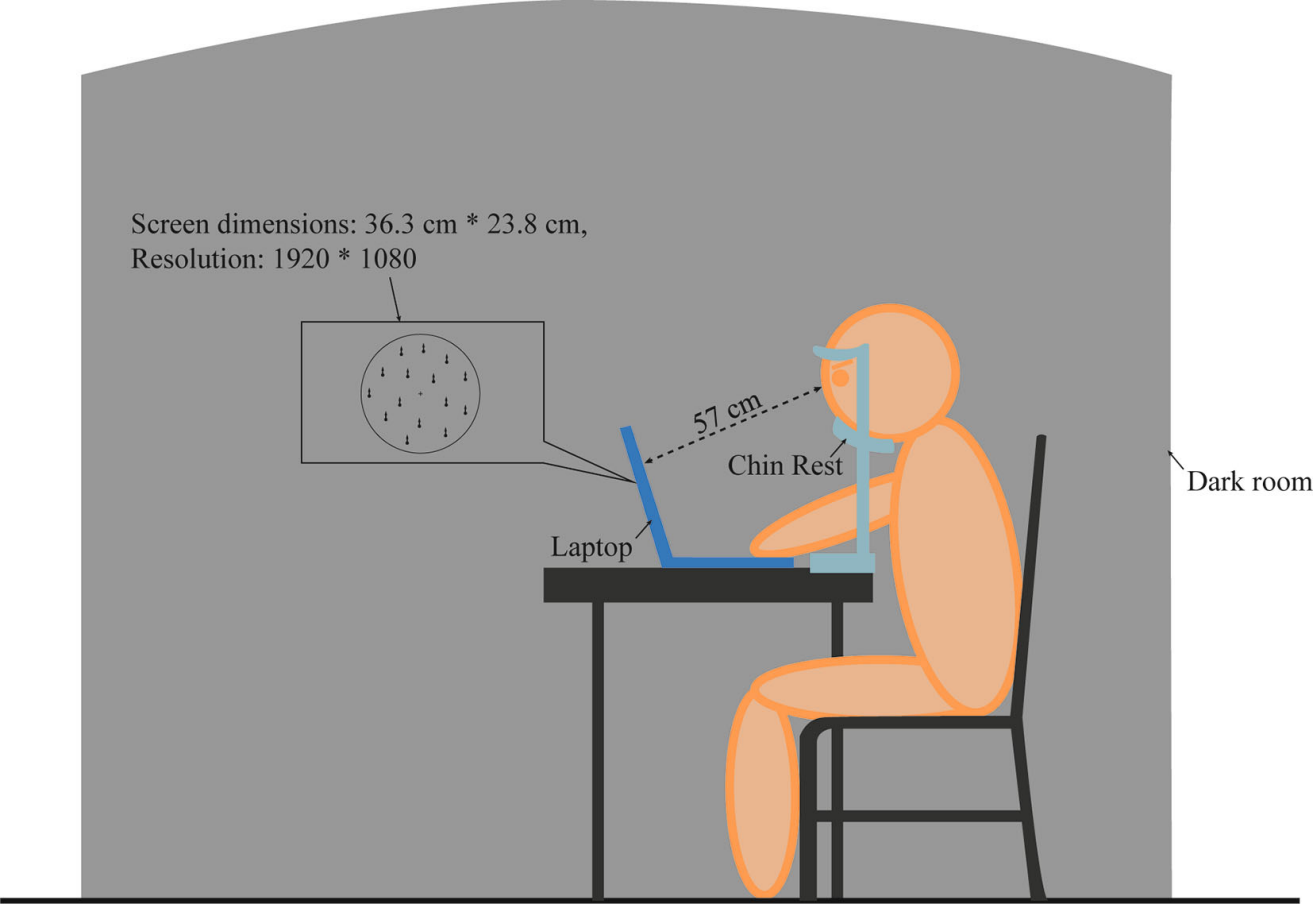
Experimental setup shows a participant adjusting the speed of the dots in the Random Dot Motion (RDM) stimulus.

During the experiment, participants adjusted the upward speed of the dots using a keyboard. The program recorded the current speed of the dots upon pressing the “Enter” key, allowing for review after the experiment. The upward movement speed of the dots ranged from 0 pixel/s (0 degrees per second) to a maximum of 1000 pixels/s (21.88 degrees per second). Bellini et al. (2023) stated that sensory environments typically use soft, dim lighting to create a tranquil atmosphere, helping to reduce anxiety and stress, and enhance comfort and relaxation, thereby positively impacting psychological and physiological health. Therefore, our experiment was conducted in a dark room designed to simulate an appropriate sensory environment ( Figure 2).

### Apparatus

A fingertip pulse oximeter (CMS50D, Contec Medical Systems Co., Ltd.) was used to monitor the participants’ heart rates throughout the experiment. We also used a stopwatch to measure participants’ subjective time perception.

### Procedures

To ensure consistency in viewing the RDM stimuli, we used a chin rest to fix the head position, maintaining 57 cm between the participant’s eyes and the screen. Participants were given control over the upward movement speed of the dots, adjusting it using arrow keys until they identified the most comfortable speed, which they confirmed by pressing the Enter key.

We employed a psychological measurement method called the method of adjustment to minimize the influence of the initial movement speed of the dots on the results. This speed varied at the beginning of each adjustment: either starting from the fastest speed (21.88 degrees per second) and gradually decreasing until the participant found the most comfortable speed, at which point they pressed the “Enter” key to record the speed; or starting from the slowest speed (0 degrees per second) and gradually increasing until they found the most comfortable speed, then recording it again. Each participant was randomly assigned to start with one of these conditions and then perform the other.

The participants repeated this process eight times, using the first two as practice trials to familiarize themselves with the procedure, and the last six to record speeds that represented their preferred speeds. We then calculated the average of the six speeds to determine the average preferred speed for each participant. This approach allowed participants to actively control the stimulus to determine their optimal comfort level. This method minimizes potential bias by using both ascending and descending adjustments, thereby providing a more accurate measurement of the participants’ preferred speed.

Based on Castellotti et al. (2022), we utilized a time-estimation task to measure each participant’s accuracy in subjective time perception. We instructed the participants to estimate the duration of a 60-second interval. Simultaneously, participants used a stopwatch to measure their actual time. They were instructed to close their eyes and initiate the timing by pressing the start button on a stopwatch. When they estimated that one minute had elapsed, they pressed the stop button. To measure the subjective time accuracy, we calculated the difference between the recorded actual time and the 60-second target interval.

Following Schandry (1981), we employed a heartbeat-tracking task to measure each participant’s interoceptive sensitivity. We instructed the participants to silently count their heartbeats over a 60-second interval while focusing on their bodies without physically checking their pulses. A fingertip pulse oximeter simultaneously recorded the heartbeat. Interoceptive sensitivity scores were calculated by determining the absolute difference between the participants’ self-reported and recorded heartbeats, providing a measure of interoceptive accuracy.

Finally, we used several measures to provide a comprehensive understanding of participants’ sensory processing and psychological states. Japanese versions of the Visual Discomfort Scale (VDS-J; Imaizumi et al., 2018) and the Trypophobia Questionnaire (TQ-J; Imaizumi et al., 2016) were used to assess participants’ levels of visual discomfort, and the simplified State-Trait Anxiety Inventory (STAI; Koizumi et al., 1998) to assess anxiety, under the DOI https://doi.org/10.5281/zenodo.14749457. These tools helped us gather detailed information about participants’ sensory and psychological profiles. The participants completed three questionnaires in a separate bright, quiet room and each questionnaire score was recorded separately for further analysis.

### Statistical analysis

SPSS statistical software was used to analyze the data. To explore the relationship between the average preferred speed (i.e., the average of the six speeds that participants identified as the most comfortable) for RDM stimulation and factors such as interoceptive sensitivity (measured by the absolute difference between perceived and actual heart rates), subjective time perception (measured by actual time and the absolute difference between estimated time and actual time), visual discomfort levels (measured by the VDS-J and TQ-J scores), and anxiety levels (measured by the STAI scores), we conducted a correlation analysis. Spearman’s rank correlation was used because of the non-normal distribution of the variables. We examined the correlation between the average preferred speed for RDM stimulation and several variables. All statistical analyses were conducted using two-tailed tests, with the significance level set at α = 0.05.

While the Spearman correlation analysis revealed significant relationships between certain variables and the Average Preferred Speed (APS), this analysis only explored the bivariate relationships between each variable and the outcome variable. To gain a more comprehensive understanding of the combined effects of multiple variables on the APS, a multiple regression analysis was required. This analysis helped identify the factors that significantly predicted APS, providing a deeper understanding of the underlying mechanisms influencing participants’ comfort levels.

## Results

### Preferred speed of RDM stimulation

The results from the method of adjustment indicated that the average preferred speed of RDM in all participants was 5.46 degrees per second ( *SD*=116.82). The preferred speed varied quite varied largely among participants. The slowest preferred speed was 1.09 degrees per second and the fastest preferred speed was 13.86 degrees per second, which was approximately 13 times faster than the slowest.

### Spearman correlation coefficients and p-values for variables relative to the average preferred speed of RDM stimulation

Spearman’s rank correlation analysis revealed significant correlations between Average Preferred Speed (APS) and several variables at the α = 0.05 significance level. A strong positive correlation ( *rs* = 0.49, 95% *CI* [.238, .681], *p* < .001) was found between the absolute difference between the Perceived and Actual Heart Rate (HRDiff ) and the APS. This indicates that individuals with a greater discrepancy between their actual and perceived heart rates, indicating lower interoceptive sensitivity, tended to perceive faster RDM stimuli as being more comfortable. This finding underscores the importance of interoceptive sensitivity in determining the most comfortable speed for the RDM.

In addition, a negative correlation ( *rs* = -0.31, 95% *CI* [-.546, -.024], *p* = .030) was observed between the APS and the Perceived Heart Rate (PHR). This indicates that individuals who perceived a higher number of heartbeats tended to find slower speeds more comfortable, suggesting a preference for less-intense sensory input when interoceptive awareness is heightened.

Furthermore, the APS was positively correlated with the Standard Deviation of the Six Recorded Speeds (SD, *rs* = 0.49, 95% *CI* [.231, .677], *p* < .001). This suggests that individuals who perceived faster speeds as more comfortable also exhibited greater variability in their preferred speeds across the six trials, indicating a preference for a wider range of RDM stimulation speeds. This finding was further supported by the significant positive correlation ( *rs* = 0.36, 95% *CI* [.080, .584], *p* = .011) observed between SD and HRDiff. This suggests that individuals with lower interoceptive sensitivity, as reflected by a larger discrepancy between actual and perceived heart rates, tended to exhibit greater variability in their preferred speeds across the six trials. This finding further highlights the complex interplay between interoceptive sensitivity and the most comfortable speed for RDM stimulation.

Finally, a positive correlation ( *rs* = 0.31, 95% *CI* [.076, .582], *p* = .028) was found between the APS and STAI scores, which reflected the participants’ anxiety levels during the experiment. This indicated that individuals with higher anxiety levels tended to perceive faster visual motion stimuli as more comfortable, suggesting that faster RDM stimulation speeds may be more effective in modulating anxiety levels.

However, other variables such as Actual Heart Rate (AHR), Subjective Time (ST), Absolute Difference between Estimated Time and Actual Time (TimeDiff ), and visual discomfort levels (measured by VDS-J and TQ-J scores) did not show significant correlations with the APS at the α = 0.05 level during the experiment. This suggests that, while interoceptive sensitivity and anxiety levels play primary roles, other factors may only influence the preferred speed to a lesser extent or in more nuanced ways. Table 1 presents the Spearman correlation coefficients and p-values for the relationships between the APS and various variables.

**Table 1.** Spearman Correlation Coefficients and P-values for variables relative to the Average Preferred Speed (APS).

Variable	Spearman Correlation Coefficient	P-Value
Standard Deviation of Six Recorded Speeds	0.49	<.001 ^**^
Actual Heart Rate	0.10	0.485
Perceived Heart Rate	-0.31	0.030 ^*^
Absolute Difference between Perceived and Actual Heart Rate	0.49	<.001 ^**^
Subjective Time	0.10	0.478
Absolute Difference between Estimated Time and Actual Time	0.20	0.162
VDS-J Scores	0.01	0.963
TQ-J Scores	0.07	0.614
STAI Scores	0.31	0.028 ^*^

* The correlation is significant at the 0.05 level (2-tailed).

** The correlation is significant at the 0.01 level (2-tailed).

### Multiple linear regression analysis

We conducted a multiple linear regression analysis to investigate the factors influencing APS. This analysis aimed to determine the extent to which various physiological and psychological factors predicted participants’ preferred RDM speeds. The following variables were selected as potential predictors of APS: PHR, HRDiff, ST, Time Diff, STAI, VDS-J, and TQ-J.

Before conducting the multiple linear regression analysis, we examined the key assumptions to ensure the robustness and reliability of the model. A scatterplot of residuals versus standardized predicted values confirmed a linear relationship between the independent and dependent variables. The Durbin–Watson statistic (DW = 2.169) indicated no autocorrelation, suggesting that the residuals were independent. Additionally, the normality of the residuals was confirmed through an analysis of skewness (0.365) and kurtosis (0.199), indicating that the residuals closely approximated a normal distribution. Finally, the homoscedasticity assumption was supported as the residual plot showed no apparent heteroscedasticity. Thus, all assumptions were met, ensuring the validity of the regression results.

We employed a stepwise regression approach with forward selection using SPSS software (IBM SPSS Statistics, Version 27, 64-bit), with an alpha level of 0.05 for entry and 0.10 for removal (probability of F-to-enter ≤ .05 and probability of F-to-remove ≥ .10), to identify the most parsimonious and explanatory model.

The stepwise regression procedure resulted in a final model that was statistically significant, *F* (3, 46) = 7.137, *p* < .001, and explained 31.8% of the variance in APS ( *R* ^2^ = .318, Adjusted *R* ^2^ = .273). Table 2 presents the detailed results of the multiple linear regression analysis.

**Table 2.** Multiple linear regression results for predicting Average Preferred Speed.

Predictor	B	SE B	β	t	p	VIF	95% CI for B
(Constant)	51.49	58.13		0.886	0.380		
STAI	9.54	2.76	0.44	3.46	0.001	1.128	3.92 to 15.17
TimeDiff	4.77	1.69	0.36	2.83	0.007	1.094	1.38 to 8.16
VDS-J	-4.06	1.68	-0.33	-2.42	0.019	1.199	-7.42 to -0.70

*Note:* N = 50. *B*, unstandardized regression coefficient; *SE B*, standard error of the coefficient; *β* = standardized coefficient; VIF, Variance Inflation Factor; STAI, State-Trait Anxiety Inventory; TimeDiff, Absolute Difference between Estimated Time and Actual Time; VDS-J, Visual Discomfort Scale Japanese version scores.

Multicollinearity was assessed using the variance inflation factor (VIF) for each predictor in the model. All VIF values were well below the threshold of 10, with a highest VIF value of 1.199. This indicates that multicollinearity is not a concern in the model, as each predictor variable exhibits low intercorrelation with the others.

The descriptive statistics for the variables used in the analysis were as follows: STAI had a mean of 21.18 (SD = 5.41), with values ranging from 10 to 31. TimeDiff had a mean of 10.53 (SD = 8.83), with a minimum of 0.31 and a maximum of 42.91. Finally, VDS-J had a mean of 13.38 (SD = 9.34), with values ranging from 1 to 55. These statistics provide a basic overview of the distribution of the independent variables used in the regression model. The dependent variable, APS, had a mean of 249.47 (SD = 116.82), with values ranging from 50.00 to 633.33. These statistics provide a comprehensive overview of the data distribution for both the independent and dependent variables used in the regression model. All the variables had valid values (N = 50).

Stepwise regression analysis revealed that these three predictive variables significantly contributed to the prediction of APS:

1. STAI: STAI scores positively predicted APS ( *B* = 9.54, *p* = .001). For every one-unit increase in STAI scores, APS increased by 9.54 pixels/second, holding other variables constant. This suggests that individuals with higher anxiety levels tend to prefer faster RDM stimulation speeds.
2. TimeDiff: TimeDiff was a significant positive predictor of APS ( *B* = 4.77, *p* = .007). For every one-second increase in the absolute difference between estimated and actual time, APS increased by 4.77 pixels/second, holding other variables constant. This suggests that individuals with less accurate time perception tend to prefer faster visual motion stimuli.
3. VDS-J: VDS-J scores negatively predicted APS ( *B* = -4.06, *p* = .019). For every one-unit increase in VDS-J scores, APS decreased by 4.06 pixels/second, holding other variables constant. This suggests that individuals with greater visual discomfort tend to prefer slower RDM stimulation speeds.

The standardized coefficients (β) indicated that STAI scores (β = 0.44) had the strongest relative influence on APS, followed by TimeDiff (β = 0.36) and VDS-J scores (β = -0.33).

The Variance Inflation Factor (VIF) values for all predictors were close to 1, indicating no serious multicollinearity issues in the final model.

Other variables, including HRDiff, PHR, AHR, ST, and TQ-J scores, did not contribute significantly to the prediction of APS in this model.

These findings provide valuable insights into the factors influencing the preferred speed for RDM stimulation. However, it is important to note that while the model explains a substantial portion of the variance in the APS (31.8%), unexplained variability remains, indicating that other factors not included in this model may also influence the preferred bubble tube speed.

## Discussion

This study aimed to investigate the relationship between various physiological and psychological factors and Average Preferred Speed (APS) during RDM stimulation. Using both Spearman’s correlation and multiple regression analyses, we identified several significant predictors of APS, providing valuable insights into the interplay between interoceptive sensitivity, subjective time perception, visual discomfort levels, anxiety levels, and the preferred speeds for bubble tubes.

The significant positive correlation between the absolute value of the Absolute Difference between Perceived and Actual Heart Rate (HRDiff ) and APS ( *rs* = 0.49, 95% *CI* [.238, .681], *p* < .001) underscores the pivotal role of interoceptive sensitivity in shaping the preferred RDM stimulation speeds. Interoceptive sensitivity and sensory processing are related via a clear mechanistic pathway. Enhanced interoceptive sensitivity enables more precise detection of internal physiological states, which, in turn, facilitates higher temporal resolution in sensory processing. This heightened internal awareness allows individuals to detect subtle physiological responses to sensory stimuli more accurately. Recent research by Grist et al. (2023) supports this connection by demonstrating that interoceptive awareness is correlated with sensory processing capabilities in neurotypical children, suggesting this relationship is fundamental to human development.

The observed preference for slower RDM speeds among individuals with higher interoceptive sensitivity stems from two key mechanisms. First, reduced speed decreases information load and prevents sensory system overload. Second, slower presentation speeds provide extended processing windows, allowing for more thorough signal integration. This pathway enhances sensory comfort in two ways: by reducing sensory overload, and by improving processing precision. Slower information presentation lowers neural stress and fatigue, while better signal-to-noise ratios and prediction accuracy promote psychological ease. Together, these factors enable effortless engagement with the sensory environment, facilitating effective tracking and integration of stimuli for an optimal sensory experience.

The positive correlation between anxiety levels and APS ( *rs* = 0.31, 95% *CI* [.076, .582] *p* = .028), as confirmed by the multiple regression analysis ( *β* = 0.44, *p* = .001), suggests that faster RDM stimulation speeds may be particularly beneficial for individuals with higher anxiety levels. This finding is consistent with that of Park and Youn (2022) who found that high-intensity visual stimuli can replenish cognitive resources and reduce anxiety. Faster speeds may induce a state of physiological arousal that counteracts the heightened arousal associated with anxiety, thereby promoting relaxation and calmness.

The multiple regression analysis not only confirmed the independent contribution of anxiety levels to the APS, but also revealed the significant influence of subjective time perception. Although not significantly correlated with APS in the correlation analysis, the Absolute Difference between Estimated Time and Actual Time (TimeDiff ) emerged as a predictor in the regression model ( *β* = 0.36, *p* = .007), suggesting that subjective time perception may interact with other factors to influence the preferred RDM stimulation speed in a complex manner. Individuals with lower subjective time-perception accuracy, in the same way as those with lower interoceptive sensitivity, may perceive faster RDM stimulation speeds as more comfortable. This finding suggests a potential link between time perception and interoceptive processing, as proposed by Di Lernia et al. (2018). It is possible that the perceived passage of time is influenced by internal bodily sensations and that individuals with heightened interoceptive awareness may experience time differently, leading them to perceive slower RDM speeds as more comfortable.

Furthermore, while visual discomfort levels did not show significant correlations with APS in the correlation analysis, the multiple regression analysis revealed some nuanced effects. Specifically, higher VDS-J scores were significantly negatively correlated with slower APS ( *β* = -0.33, *p* = .019). These results indicate that although visual discomfort may not independently predict the APS, it contributes to the overall model of the preferred speed. This suggests that individuals with higher visual discomfort levels perceive slower speeds as more comfortable. This finding aligns with that of Penacchio et al. (2023) who suggested that slower visual stimulus speeds can alleviate discomfort in individuals with visual sensitivity. This emphasizes the importance of tailoring the sensory stimuli to individual comfort levels to maximize the therapeutic benefits of RDM stimulation.

These insights contribute to our understanding of the complex interplay between physiological and psychological factors in determining the preferred RDM stimulation speed and suggest potential pathways for designing sensory interventions. Our findings showed that participants’ preferred RDM stimulation speeds varied according to their physiological and psychological factors, underscoring the need for personalized design in bubble tubes and sensory rooms.

## Conclusions

This study provides valuable insights into the complex interplay between the physiological and psychological factors that influence preferred RDM stimulation speeds. Our findings reveal that interoceptive sensitivity, subjective time perception, visual discomfort, and anxiety are significant predictors.

These results demonstrate that individuals with lower interoceptive sensitivity tend to prefer faster RDM stimulation speeds, possibly as a compensatory mechanism for reduced internal bodily awareness. This relationship between interoceptive sensitivity and the preferred speed underscores the importance of considering individual physiological differences in bubble tubes and sensory rooms.

Anxiety levels emerged as a significant factor, with participants’ anxiety levels significantly and positively correlated with the preferred RDM speed. This finding suggests that faster and more intense visual stimuli may have therapeutic applications for individuals with anxiety disorders. Saiu and Grosso (2022) demonstrated that combined audio-visual stimulation significantly reduced anxiety levels, systolic blood pressure, and heart rate in patients undergoing surgery, supporting the potential of multi-sensory interventions in anxiety management.

The subjective time perception ability was also related to the preferred RDM stimulation speed, with participants with lower accuracy in time perception tending to prefer faster speeds. Previous research has shown that interoceptive focus significantly influences subjective time perception, with heightened interoceptive awareness amplifying the time-dilating effects of fear and the time-accelerating effects of amusement ( Pollatos et al., 2014). This, in turn, may have affected participants’ perceptions of comfort in response to RDM stimulation speeds.

Although the visual discomfort levels were not independently correlated with the preferred speed in our Spearman’s correlation analysis, they emerged as significant predictors in our multiple regression analysis. Specifically, participants with higher visual sensitivity tended to prefer slower speeds. This finding highlights the importance of designers considering individual levels of visual discomfort when designing visual stimuli in bubble tubes or sensory rooms and offering tailored sensory experiences that cater to each person’s diverse sensory needs.

As Ziegler (2015) noted in her research on multi-sensory design in healthcare settings, environments that allow users to customize sensory inputs contribute to stress reduction and improved well-being. Garzotto et al. (2019) developed extensible multilayer software and hardware platform to connect and manage different devices in a sensory room, thereby enabling therapists to fully customize activities in multi-sensory environments for different children. The findings of this study support the idea that tailored sensory environments, such as those incorporating adjustable bubble tube speeds, can enhance the therapeutic outcomes for individuals with varying sensory hypersensitivities and psychological states.

Building on our previous research ( Su et al., 2024), which demonstrated that sensory-hypersensitive individuals have greater variations in their preferences for wallpaper colors and patterns, this study further underscores the necessity for customizable sensory rooms tailored to each person’s unique sensory needs.

Future research should explore the potential therapeutic applications of tailored bubble tube speed stimulation to reduce anxiety. It is also crucial to investigate the intrinsic relationships among variables such as interoceptive sensitivity, subjective time perception, anxiety levels, and visual discomfort. Additionally, conducting longitudinal studies to examine how participants’ preferred speeds change over time or with repeated exposure to the same visual stimuli would provide valuable insights.

In conclusion, this study enhances our understanding of the factors influencing preferred bubble tube speeds and lays the groundwork for the development of more personalized and effective sensory interventions to improve outcomes for individuals with diverse sensory needs.

### Ethical considerations

This study recruited 50 healthy participants (27 females) aged 22 to 35 years (M = 25.94, SD = 2.74) from the University of Tsukuba between December 25, 2023, and March 22, 2024. The research was conducted in accordance with the principles of the Declaration of Helsinki and was approved by the Institutional Review Board (IRB) of the Institute of Art and Design, the University of Tsukuba (IRB No. [GEI021-15]) on March 22, 2022. Prior to participation, all participants provided written informed consent. To ensure privacy, all participant data were anonymized, and no identifying information was collected.

## Consent

Written informed consent for publication of the participants details was obtained from the participants.

## Data Availability

Zenodo: Preferred Bubble Tube Speed and Physiological–Psychological Factors, DOI: 10.5281/zenodo.14633771 ( Su, A., 2025b). This project contains the following underlying data: Preferred bubble tube speed and physiological–psychological factors.xlsx – Contains measurements from an experiment investigating the relationship between preferred bubble tube speed and physiological–psychological factors, including data collected from 50 participants. Data are available under the terms of the Creative Commons Attribution 4.0 International license (CC-BY 4.0). Zenodo: Checklist for Preferred Bubble Tube Speed and Physiological–Psychological Factors, DOI: 10.5281/zenodo.14633918 ( Su, A., 2025a). This project contains the following reporting guideline document:
• STROBE-checklist-Su_Anjie.doc – A completed STROBE checklist for this study. Data are available under the terms of the Creative Commons Attribution 4.0 International license (CC-BY 4.0). All experimental materials, including psychometric scales and experimental software, are openly accessible to ensure reproducibility. Zenodo: Scales used to assess participants’ levels of visual discomfort and anxiety, DOI: https://doi.org/10.5281/zenodo.14770842 ( Su, A., 2025e). This project contains the following underlying data:
• The Simplified State-Trait Anxiety Inventory (STAI) – Japanese version.pdf – A validated scale assessing state and trait anxiety, used in this study. The Simplified State-Trait Anxiety Inventory (STAI) – English version.pdf – The English version of the STAI scale for reference. • The Trypophobia Questionnaire – Japanese version (TQ-J).pdf – A psychometric tool assessing trypophobia symptoms, adapted into Japanese. • The Trypophobia Questionnaire – English version.pdf – The original version of the Trypophobia Questionnaire for reference. • The Visual Discomfort Scale – Japanese version (VDS-J).pdf – A validated scale measuring visual discomfort, adapted into Japanese. • The Visual Discomfort Scale – English version.pdf – The original version of the Visual Discomfort Scale for reference. These materials are licensed under the Creative Commons Attribution 4.0 International (CC-BY 4.0) license, permitting unrestricted use, distribution, and reproduction, provided the original authors and the present archived datasets are appropriately cited.

## Software availability statement

• Source Code GitHub Repository: https://github.com/Lem0n-SAJ/RDM_Program_Su
• Zenodo DOI (Source Code): https://doi.org/10.5281/zenodo.14795461
• Zenodo DOI (Windows Executable): https://doi.org/10.5281/zenodo.14795194

The software is distributed under the MIT License.
