# Exploring the relationship between preferred bubble tube speeds in sensory rooms and physiological–psychological factors: A study on interoceptive sensitivity, subjective time perception, visual discomfort levels, and anxiety levels

**DOI:** 10.12688/f1000research.161090.2

**Published:** 2026-03-27

**Authors:** Anjie Su, Junyi Shen, Shinichi Koyama

**Affiliations:** 1 Doctoral Program in Design, University of Tsukuba, Tsukuba, Ibaraki Prefecture, Japan; 2 Institute of Art and Design, University of Tsukuba, Tsukuba, Ibaraki Prefecture, Japan

**Keywords:** Sensory Hypersensitivity, Sensory Room, Bubble Tubes, Interoceptive Sensitivity, Subjective Time Perception, Visual Discomfort, Anxiety

## Abstract

**Background:**

This study examined how preferred bubble-tube motion speeds in sensory rooms relate to individual physiological and psychological characteristics, including interoceptive sensitivity, subjective time perception, visual discomfort, and anxiety levels.

**Methods:**

Fifty adult participants took part in a controlled laboratory experiment using a method-of-adjustment procedure to select their most comfortable motion speed for a simulated bubble tube, presented as an upward-moving Random Dot Motion (RDM) stimulus. Subjective time perception was evaluated using a 60-second time-estimation task, and interoceptive sensitivity was measured via a heartbeat-tracking task. Visual discomfort and anxiety were assessed using the Japanese versions of the Visual Discomfort Scale (VDS-J), Trypophobia Questionnaire (TQ-J), and State-Trait Anxiety Inventory (STAI).

**Results:**

The results from the method of adjustment indicated that the preferred speed varied widely, from 1.09 to 13.86 degrees per second. Spearman’s correlation analysis revealed that higher interoceptive awareness was associated with a preference for slower speeds, whereas higher anxiety levels were associated with a preference for faster speeds. In addition, multiple regression analysis showed that subjective time-perception accuracy and visual discomfort levels were significant predictors of participants’ preferred RDM speeds.

**Conclusion:**

The results indicate that interoceptive sensitivity, subjective time perception, visual discomfort, and anxiety levels play significant roles in determining preferred RDM stimulation speeds. These findings highlight the importance of considering individual differences in physiological and psychological states when designing therapeutic sensory environments, such as sensory rooms and bubble tubes, to support comfort, well-being, and therapeutic outcomes.

## Introduction

Sensory overload is a common experience among individuals with sensory hypersensitivity, in which everyday stimuli such as light ( Lucker, 2013), noise ( Khalfa et al., 2004; Landon et al., 2016), and touch ( Blakemore et al., 2006) can become overwhelming. Individuals may find it challenging to navigate routine environments ( Lucker, 2013), which can affect their ability to function effectively in daily life ( Kim et al., 2000). Researchers and practitioners have developed various methods and tools to provide sensory support ( Koegel et al., 2004). These approaches aim not only to reduce stress associated with sensory overload ( Mazurek et al., 2013) but also to enhance quality of life and agency within everyday environments ( Hannant et al., 2016). Studies have shown that sensory hypersensitivity can significantly influence behavior and emotional experiences and is associated with heightened distress responses ( Engel-Yeger et al., 2016). Consistent with this, work on sensory over-responsivity (SOR) in autism demonstrates robust links with anxiety across development, including theoretical and longitudinal evidence that SOR and anxiety can exacerbate one another ( Green & Ben-Sasson, 2010; Green et al., 2012). More recent studies further link sensory reactivity differences to anxiety subtypes in autistic children and to perceived causal relations in autistic adults ( MacLennan et al., 2020, 2021; Verhulst et al., 2022).

Designed to provide a controlled environment that supports self-regulation and relaxation, sensory rooms are typically equipped with assistive technologies and specialized elements. A prominent example of the Snoezelen room, a multisensory environment developed in the Netherlands in the 1970s ( Hulsegge & Verheul, 2005). Snoezelen rooms combine sensory experiences to create a soothing atmosphere in which users can explore stimuli at their own pace, thereby promoting relaxation and reducing stress ( Hulsegge & Verheul, 2005). These environments are particularly beneficial in psychiatric inpatient care, where they provide sensory-accessible spaces that help individuals navigate environmental stressors. Research has shown that sensory rooms, including Snoezelen rooms, significantly enhance emotional well-being and reduce distress, especially among individuals who experience heightened distress responses or anxiety ( Haig & Hallett, 2023; West et al., 2017). They also serve as valuable tools for self-management and offer alternatives to more restrictive practices ( Barbic et al., 2019). In forensic mental health settings, these rooms have been shown to reduce inpatient stress, support recovery, and enhance overall lived experience within facilities ( Wiglesworth & Farnworth, 2016).

Among commonly used sensory-room equipment, bubble tubes often serve as a central element, providing visual and auditory stimulation. These tall, water-filled cylinders produce continuously rising bubbles via an internal air pump and are often paired with color-changing LED lights, supporting visual tracking, color engagement, and gentle auditory cues. The continuous movement and changing colors of the bubbles can soothe and attract attention ( Fujisawa, 2021). Bubble tubes are frequently described as calming for autistic people and others with sensory processing differences; observational studies indicate that they are among the most popular items in multisensory environments ( Unwin et al., 2024).

However, the precise mechanisms by which bubble tubes promote relaxation remain poorly understood. Research on sensory modulation in psychiatric inpatient settings and multisensory environments suggests that low-demand, user-controlled multisensory input can support arousal regulation and reduce distress ( Champagne & Stromberg, 2004; Sutton et al., 2013; Scanlan & Novak, 2015). From a sensory-integration perspective, foundational models emphasize individual differences in sensory thresholds and self-regulatory capacity that likely shape responses to such environments ( Dunn, 2001; Lane et al., 2019). Syntheses of evidence across multisensory environment implementations report generally positive within-session effects on calm and engagement but variable outcomes across populations and programs, underscoring the need to identify person-level moderators ( Breslin et al., 2020; Van Weert et al., 2005; Haig & Hallett, 2023).

We focused on four constructs that plausibly shaped comfort during visual stimulation. Interoception can be described in at least three related facets: (i) accuracy—objective performance on tasks indexing detection of internal bodily signals; (ii) sensibility—self-reported beliefs and attention toward internal sensations; and (iii) awareness—metacognitive insight into one’s interoceptive performance ( Garfinkel et al., 2015; Critchley & Garfinkel, 2017). In the present study, we focused on interoceptive accuracy, assessed using an objective heartbeat-tracking task, because objective indices are most directly comparable with behavioral preferences measured in our speed-selection paradigm ( Garfinkel et al., 2015). Perception of internal bodily signals (e.g., heartbeat) supports monitoring and adjustment of emotional and physiological states, shaping responses to external sensory input ( Garfinkel & Critchley, 2013; Pollatos et al., 2012; Tanaka et al., 2021).

Subjective time perception, shaped by attention, emotion, and sensory input, can influence how dynamic visual stimuli are experienced and may covary with interoceptive processes; timing errors may bias preferred motion speeds toward what feels subjectively “right” ( Yabe & Yamada, 2023). Time processing in the seconds range recruits the insular cortex and covaries with interoceptive accuracy, such that individuals with more precise detection of cardiac signals tend to show more accurate duration judgments ( Meissner & Wittmann, 2011; Vicario et al., 2020). Affective states also modulate temporal judgments: meta-analytic and experimental work indicates that fear and anxiety can distort perceived duration via changes in arousal and attentional allocation, with anxiety in particular biasing underestimation in threat-of-shock paradigms ( Droit-Volet & Meck, 2007; Sarigiannidis et al., 2020). Given that bubble tubes are intended to enhance well-being and are used by people with sensory processing differences, it is pertinent to examine how interoceptive accuracy and time perception relate to preferred bubble-tube speeds.

Visual discomfort can also shape responses to bubble-tube stimulation. Individuals reporting higher visual discomfort often perform visual tasks more slowly, and uncomfortable visual patterns are associated with stronger yet less frequent neural responses, consistent with less efficient coding ( Conlon & Humphreys, 2001; Wilkins, 2016). Accordingly, the speed of visual motion may either alleviate or exacerbate discomfort, making it a design-relevant parameter in sensory rooms. Visual discomfort is reliably elicited by images whose spatial statistics deviate from natural 1/f structure—typically with excess mid-spatial-frequency energy—and by patterns that provoke pattern glare (e.g., high-contrast stripes), with elevated susceptibility in migraine and related conditions ( Evans & Stevenson, 2008; Penacchio & Wilkins, 2015). Analogous principles apply in the temporal domain: flicker and temporal profiles that depart from natural temporal statistics increase discomfort, and adaptation can systematically shift both perceived temporal sharpness and discomfort ( Yoshimoto et al., 2019). These observations motivate treating motion speed as a design-relevant parameter that can either alleviate or exacerbate discomfort in sensory rooms.

Anxiety is likewise important. Elevated state anxiety is linked to heightened sensitivity to environmental cues and greater distress under sensory load ( Åhs et al., 2013), and greater sensory-processing sensitivity is associated with higher anxiety, depression, and stress, particularly when mindfulness and acceptance are low ( Bakker & Moulding, 2012). Research on SOR in autism indicates sustained associations with anxiety, including bidirectional influences observed longitudinally in toddlers ( Green et al., 2012) and systematic links to anxiety subtypes in childhood as well as perceived causal pathways in adults ( MacLennan et al., 2020, 2021; Verhulst et al., 2022; Green & Ben-Sasson, 2010). In the present study, we assessed both state anxiety (a transient, context-dependent anxious affect) and trait anxiety (a relatively stable dispositional tendency) using the simplified State-Trait Anxiety Inventory ( Spielberger, 1983; Koizumi et al., 1998). Conceptually, state anxiety primarily indexes momentary arousal that can shift sensory gating, whereas trait anxiety has been linked to broader differences in attentional control that may heighten stimulus-driven processing ( Eysenck et al., 2007). Taken together, these considerations underscore the relevance of interoceptive accuracy, subjective time perception, visual discomfort, and anxiety in understanding individual variation in preferred bubble-tube speed.

Although we did not recruit a clinically characterized cohort, adjacent work indicates that person-level sensory profiles and user control of sensory inputs shape engagement and calm in multi-sensory environments ( Dunn, 2001; Lane et al., 2019; Scanlan & Novak, 2015; Champagne & Stromberg, 2004; Unwin et al., 2022, 2024). In autistic populations, reviews document distinctive motion and multisensory processing that can shift preferred parameters of dynamic visual input ( Robertson & Baron-Cohen, 2017). These observations motivate treating bubble-tube motion speed as a design-tunable parameter and considering person-level characteristics that plausibly modulate comfort, without presupposing clinical diagnosis.

We used an upward-moving random dot motion (RDM) display to isolate the motion-speed component of bubble-tube viewing while minimizing confounds from color, texture, and form. RDM/RDK paradigms are canonical tools for probing global motion, with well-established neural underpinnings in primate area MT/V5 and tight psychophysics–neurophysiology links ( Newsome & Paré, 1988; Britten et al., 1992; Salzman et al., 1992). In neurodiversity research, RDM tasks are widely used to assess global motion processing in autistic participants, with mixed but convergent evidence for small group-level differences and strong parameter control across studies (e.g., Milne et al., 2002; Koldewyn et al., 2010; Van der Hallen et al., 2019; see also Robertson & Baron-Cohen, 2017). This makes RDM an appropriate, translationally useful proxy for bubble-tube motion when the design-tunable variable of interest is speed.

In this study, we tested whether interoceptive accuracy, subjective time perception, anxiety, and visual discomfort levels jointly predict preferred stimulation speed. This approach provides design-relevant estimates that can be translated into adjustable settings for sensory-room equipment.

## Methods

### Participants

The sample size was determined through a priori power analysis using G*Power (version 3.1). Based on the consideration of three key parameters—a large effect size (r = 0.50) following Cohen’s (1988) guidelines to reflect the anticipated substantive and meaningful association between variables, a conventional significance level ( *α* = 0.05) to maintain an acceptable balance between Type I error rates and the ability to detect true effects, and a statistical power (1- *β* = 0.80) to ensure an 80% likelihood of detecting a true effect if one existed—G*Power recommended a minimum sample size of 42 participants. To account for potential data loss or participant dropout, we recruited 50 participants to enhance the robustness and reliability of the statistical analyses.

Participants were not screened for clinical diagnoses (e.g., autism, anxiety, or mood-related conditions), and psychotropic medication was not recorded. Our aim was to model inter-individual differences in a non-clinical adult sample rather than to estimate effects by diagnostic subgroup; we did not solicit diagnostic disclosures to minimize privacy burden and because our predictors (interoceptive accuracy, time-estimation error, state anxiety, visual discomfort) are non-diagnostic mechanistic indices relevant to design-tunable parameters.

We recruited 50 participants (27 females) aged 22–35 years (M = 25.94, SD = 2.74) from the University of Tsukuba between December 25, 2023, and March 22, 2024. Prior to participation, all participants provided written informed consent as approved by the Institutional Review Board (IRB) of the Institute of Art and Design, the University of Tsukuba (IRB No. [GEI021-15]). On the day of the experiment, participants were asked to abstain from alcohol, caffeine, and cigarettes to support consistency of physiological state across participants. Participants reported adequate sleep and typical or corrected-to-typical vision.

### Stimuli

**Figure 1.**
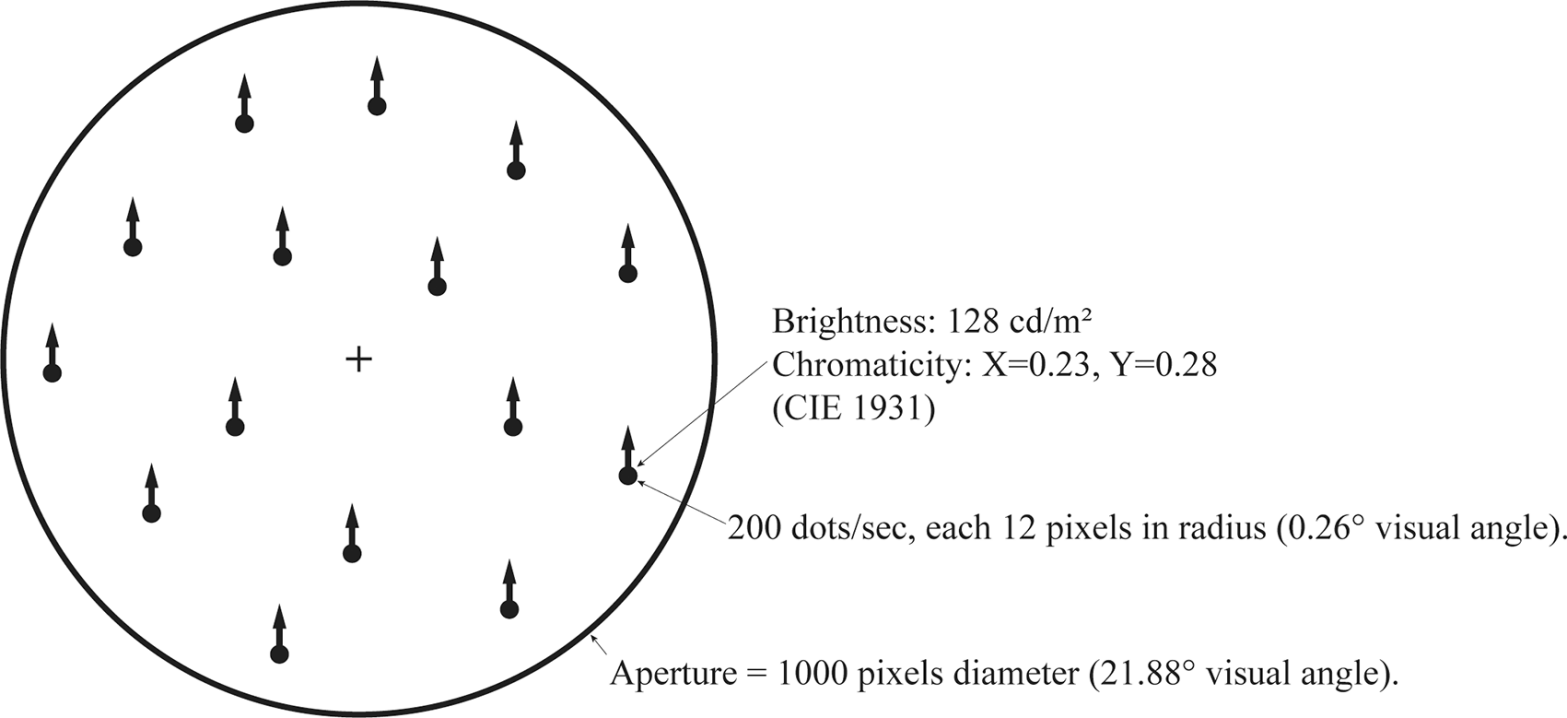
Schematics of the Random Dot Motion (RDM) stimulus.

We used a Random Dot Motion (RDM) stimulus program developed using the Flutter SDK. The source code (v1.0) is available under the MIT License on Zenodo ( Su, 2025; https://doi.org/10.5281/zenodo.14795461), with a Windows executable build concurrently archived ( Su, 2025d; https://doi.org/10.5281/zenodo.14795194). The program ran on a Lenovo laptop (screen dimensions: 36.3 cm × 23.8 cm, resolution: 1920 × 1080, model number: 115423562). Our RDM program generated dots that moved vertically upward within a circular aperture centered on the screen ( Figure 1). All dots moved at the same speed, simulating the upward movement of the bubbles in the tube. This circular area had a diameter of 1000 pixels, corresponding to a visual angle of 21.88°. A white fixation cross was placed at the center of the circular area. This helped ensure that participants maintained fixation on a specific location and sustained visual attention, facilitating detection of subsequent stimuli. During the experiment, the program randomly generated 200 dots per second within a circular area. Each dot had a radius of 12 pixels, corresponding to a visual angle of 0.26°. All dots had the same brightness and chromaticity, set at 128 cd/m ^2^, X = 0.23, Y = 0.28 in the CIE 1931 color space, ensuring consistent visual presentation.

The RDM stimulus allowed parametric control over upward motion speed (°/s) while holding dot density, lifetime, and luminance statistics constant, thereby aligning the laboratory manipulation with the adjustable bubble-rise rate used in sensory-room equipment. This choice is grounded in extensive basic-science validation of RDM for motion perception and in its widespread use to characterize motion processing in autistic samples, facilitating comparison with prior neurodiversity research ( Newsome & Paré, 1988; Britten et al., 1992; Salzman et al., 1992; Milne et al., 2002; Koldewyn et al., 2010; Van der Hallen et al., 2019).

**Figure 2.**
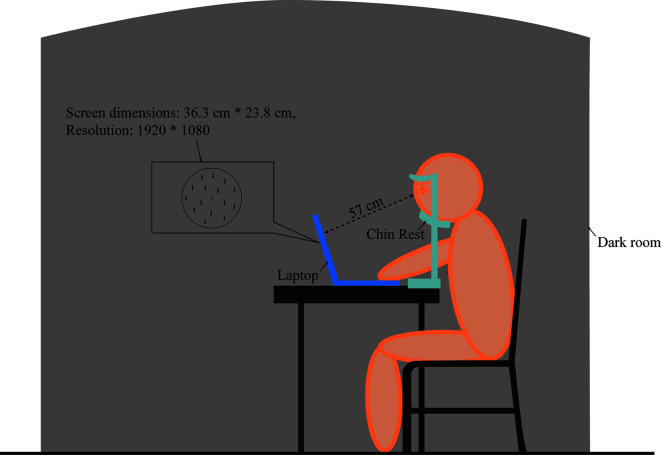
Experimental setup shows a participant adjusting the speed of the dots in the Random Dot Motion (RDM) stimulus.

During the experiment, participants adjusted the upward speed of the dots using a keyboard. The program recorded the current dot speed when the “Enter” key was pressed, allowing for later review. The upward motion speed ranged from 0 pixel/s (0°/s) to 1000 pixels/s (21.88°/s). Bellini et al. (2023) reported that sensory environments typically use soft, dim lighting to create a tranquil atmosphere that supports comfort, relaxation, and reduced stress. Therefore, the experiment was conducted in a darkened room designed to simulate a sensory-room environment ( Figure 2).

### Apparatus

A fingertip pulse oximeter (CMS50D, Contec Medical Systems Co., Ltd.) was used to monitor the participants’ heart rates throughout the experiment. We also used a stopwatch to measure participants’ subjective time perception.

### Procedures

To ensure consistent viewing of the RDM stimuli, a chin rest was used to maintain a viewing distance of 57 cm between the participant’s eyes and the screen. Participants were given control over the upward movement speed of the dots, adjusting it using arrow keys until they identified the most comfortable speed, which they confirmed by pressing the Enter key.

A consolidated summary of all measures is presented in Tables 1A (experimental tasks) and 1B (questionnaires), including key settings, scoring, and validation references.

**Table 1A.** Experimental tasks.

Construct	Task	Key settings	Scoring	Validation refs
Preferred stimulation speed	Method of Adjustment with upward RDM	6 runs; ascending & descending starts; aperture 21.88°; 200 dots/s	Mean of 6 speeds (deg/s)	Wier et al., 1976
Subjective time perception	60-s time-estimation	Eyes closed; self start/stop stopwatch	|recorded − 60|in seconds	Castellotti et al., 2022
Interoceptive accuracy	Heartbeat-tracking	60 s; no palpation; pulse oximeter	|counted−recorded|	Schandry, 1981

**Table 1B.** Questionnaires.

Construct	Instrument	Items/Scale	Scoring	Validation refs
Visual discomfort (General)	VDS-J	23 items; 0–3	Sum/mean (higher = more)	Imaizumi et al., 2018
Visual discomfort (Trypophobia)	TQ-J	17 items; 1–5	Sum/mean	Imaizumi et al., 2016
Anxiety (State & Trait)	STAI-Y (JP short form)	4-point; Y-1 & Y-2	Separate state/trait sums	Koizumi et al., 1998

**Open science note.** All materials required to reproduce the stimuli, measures, and analyses are openly available; see the Data, code, and materials availability section for persistent links (DOIs).

We used a method-of-adjustment procedure with an upward random-dot motion (RDM) display to isolate the motion-speed component of bubble-tube viewing while minimizing color/texture/form confounds. Each participant completed eight runs (two practice, six recorded) with randomized ascending (0→21.88°/s) and descending (21.88→0°/s) starting speeds. Participants adjusted speed until it felt most comfortable and pressed “Enter” to record the value. The primary dependent variable was the mean of the six recorded speeds (°/s). This classical method-of-adjustment approach is standard in psychophysics and supports precise parameter control with RDM.

Following Castellotti et al. (2022), participants completed a 60-s time-estimation task with eyes closed, starting and stopping a stopwatch when they judged that 60 s had elapsed. We computed absolute error (|recorded − 60|in seconds).

We administered the heartbeat-tracking task following Schandry (1981). Participants silently counted heartbeats during a 60-s interval without palpation, while a fingertip pulse oximeter recorded actual beats. Interoceptive accuracy was computed as|counted − recorded|(lower values indicate better accuracy).

General tendency to visual discomfort was assessed using the Japanese Visual Discomfort Scale (VDS-J; 23 items, 0–3); the Japanese version shows a unidimensional Rasch structure, Rasch person reliability of approximately 0.82, good test–retest stability, and construct validity (higher scores reported in migraine) ( Imaizumi et al., 2018). Discomfort to clustered patterns was assessed with the Japanese Trypophobia Questionnaire (TQ-J; 17 items, 1–5), which demonstrates a one-factor structure and adequate internal consistency and test–retest reliability in Japanese adults ( Imaizumi et al., 2016).

We measured both state and trait anxiety using the simplified State-Trait Anxiety Inventory (STAI; Koizumi et al., 1998) to assess anxiety.

These scales provided detailed information about participants’ sensory and psychological profiles. All participants completed three questionnaires in a separate bright and quiet room, and each questionnaire score was recorded separately for further analysis. We summarized all measures in Tables 1A, 1B and the essential task settings and scoring rules are reported below.

### Statistical analysis

To explore the relationship between the average preferred speed (APS) (i.e., the mean of the six speeds that participants identified as most comfortable) for RDM stimulation and factors such as interoceptive sensitivity (measured by the absolute difference between perceived and actual heart rates), subjective time perception (measured by actual time and the absolute difference between estimated time and actual time), visual discomfort levels (measured by the VDS-J and TQ-J scores), and anxiety levels (measured by the STAI scores), we conducted correlation analyses. Prior to inference, we screened the data for entry errors and outliers and inspected Q–Q plots and Shapiro–Wilk tests; several variables (e.g. Absolute Difference between Estimated Time and Actual Time, Absolute Difference between the Perceived and Actual Heart Rate) departed from normality. Accordingly, Spearman’s rank correlations were used for bivariate associations. We computed Spearman’s *r*s and 95% confidence intervals (CI) via bootstrap (5,000 resamples). We examined the correlation between the APS for RDM stimulation and several variables. All statistical analyses were conducted using two-tailed tests, with the significance level set at α = 0.05.

While the Spearman correlation analysis revealed significant relationships between certain variables and the APS, this analysis examined only bivariate associations. To examine the combined effects of multiple variables on APS, we conducted a multiple regression analysis. This analysis identified factors that significantly predicted APS, providing a deeper understanding of the underlying mechanisms influencing participants’ comfort levels.

All analyses were conducted using IBM SPSS Statistics v27 (64-bit) via the graphical interface; no custom code was written. To facilitate reuse, we provide de-identified data files (.xlsx and .sav), complete SPSS output files (.spv) containing the exact procedures and parameter settings and a step-by-step analysis recipe (PDF) that enumerates each transformation and test from raw variables to the reported tables and figures.

## Results

### Descriptive statistics

Table 2 summarizes means, standard deviations, ranges, and distribution shape (skewness, kurtosis) for all variables (N = 50). APS showed wide inter-individual variability (M = 249.47, SD = 116.82; range = 50.00–633.33). TimeDiff and the visual-discomfort measures (VDS-J, TQ-J) were positively skewed, whereas other variables were approximately symmetric; accordingly, bivariate associations used Spearman’s rs, and regression inference relied on residual diagnostics.

**Table 2.** Descriptive statistics for all variables.

Variable	N	Mean	SD	Range	Skewness	Kurtosis
APS	50	249.47	116.82	50.00–633.33	0.95	1.40
AHR	50	79.14	9.20	60.00–102.00	0.28	−0.29
PHR	50	63.96	16.82	33.00–100.00	0.30	−0.62
HRDiff	50	17.46	13.66	1.00–44.00	0.69	−0.66
ST	50	67.07	11.84	39.56–102.91	0.44	0.78
TimeDiff	50	10.53	8.83	0.31–42.91	1.37	2.56
VDS-J	50	13.38	9.34	1.00–55.00	2.00	6.83
TQ-J	50	28.82	12.50	17.00–63.00	1.41	1.05
STAI total	50	21.18	5.41	10.00–31.00	−0.24	−0.93

### Spearman correlation coefficients and p-values for variables relative to the average preferred speed of RDM stimulation

Spearman’s rank correlation analysis revealed significant correlations between APS and several variables at *α* = 0.05. A strong positive correlation ( *rs* = 0.49, 95% *CI* [.267,.660], *p* < .001) was found between the Absolute Difference between the Perceived and Actual Heart Rate (HRDiff) and the APS. This indicates that individuals with a greater discrepancy between their actual and perceived heart rates, reflecting lower interoceptive sensitivity, tended to perceive faster RDM stimuli as more comfortable. This finding underscores the relevance of interoceptive sensitivity in preferred RDM speed.

In addition, a negative correlation ( *rs* = -0.31, 95% *CI* [-.555, -.025], *p* = .030) was observed between the APS and the Perceived Heart Rate (PHR). This indicates that individuals who perceived a higher number of heartbeats tended to find slower speeds more comfortable, suggesting a preference for less-intense sensory input when interoceptive awareness is heightened.

Furthermore, the APS was positively correlated with the Standard Deviation of the Six Recorded Speeds (SD, *rs* = 0.49, 95% *CI* [.204, .709], *p* < .001). This suggests that individuals who preferred faster speeds also exhibited greater variability across the six trials, indicating a wider range of preferred RDM speeds. This finding was further supported by the significant positive correlation ( *rs* = 0.36, 95% *CI* [.108,.560], *p* = .011) observed between SD and HRDiff. This suggests that participants with lower heartbeat-tracking accuracy tended to show greater variability in preferred speeds across the six trials. This finding further highlights the association between interoceptive sensitivity and preferred RDM speed.

Finally, a positive correlation ( *rs* = 0.36, 95% *CI* [.060, .601], *p* = .011) was found between APS and STAI scores, reflecting participants’ anxiety levels during the experiment. This indicates that participants with higher STAI scores tended to perceive faster visual motion stimuli as more comfortable, suggesting that faster RDM stimulation speeds may be more effective in modulating anxiety levels.

However, Actual Heart Rate (AHR), Subjective Time (ST), Absolute Difference between Estimated Time and Actual Time (TimeDiff), and visual discomfort (VDS-J and TQ-J scores) did not show significant correlations with APS at *α* = 0.05. This suggests that while interoceptive sensitivity and anxiety levels play primary roles, other factors may influence the preferred speed to a lesser extent or in more nuanced ways. Table 3 presents the Spearman correlation coefficients and *p*-values for the relationships between the APS and various variables.

**Table 3.** Spearman Correlation Coefficients and P-values for variables relative to the Average Preferred Speed (APS).

Variable	Spearman Correlation Coefficient	P-Value
Standard Deviation of Six Recorded Speeds	0.49	<.001 ^**^
Actual Heart Rate	0.10	0.485
Perceived Heart Rate	-0.31	0.030 ^*^
Absolute Difference between Perceived and Actual Heart Rate	0.49	<.001 ^**^
Subjective Time	0.10	0.478
Absolute Difference between Estimated Time and Actual Time	0.20	0.162
VDS-J Scores	0.01	0.963
TQ-J Scores	0.07	0.614
STAI Scores	0.31	0.028 ^*^

* The correlation is significant at the 0.05 level (2-tailed).

** The correlation is significant at the 0.01 level (2-tailed).

### Multiple linear regression analysis

We specified a single multiple-regression model for APS with a limited, theory-driven set of candidate predictors (PHR, HRDiff, ST, TimeDiff, VDS-J, TQ-J, STAI). Because inference targeted the model as a whole and the number of pre-specified predictors was modest, we did not apply a formal multiplicity correction (two-sided *α* = .05). Instead, we emphasize effect sizes and 95% CI, consistent with guidance cautioning against routine Bonferroni-type adjustments in planned analyses ( Rothman, 1990; Perneger, 1998). To obtain a parsimonious subset we used forward stepwise selection (F-to-enter ≤ .05; F-to-remove ≥ .10) and interpret selected predictors cautiously given known limitations of automated selection ( Babyak, 2004; Derksen & Keselman, 1992; Whittingham et al., 2006). Analyses were conducted in SPSS (v27, 64-bit).

Model assumptions were checked and met, including linearity (residuals vs. fitted), independence (Durbin–Watson = 2.17), normality (skew = 0.37, kurtosis = 0.20), and homoscedasticity (residual plots).

The final model was significant, F (3, 46) = 7.14, *p* < .001, explaining 31.8% of the variance in APS (R ^2^ = .318, adjusted R ^2^ = .273). The detailed coefficients are presented in Table 4.

Multicollinearity was assessed using the variance inflation factor (VIF) for each predictor in the model. All VIF values were well below the threshold of 10, with a highest VIF value being 1.199. This indicates that multicollinearity is not a concern in the model, as each predictor variable exhibits low intercorrelation with the others. Additionally, no multiplicity correction was applied; *p* values are two-sided and should be interpreted descriptively alongside effect sizes and *CI*s ( Rothman, 1990; Perneger, 1998).

Our linear regression analysis revealed that three predictors significantly contributed to APS:

• STAI: STAI scores positively predicted APS. This suggests that participants with higher STAI scores tend to prefer faster RDM stimulation speeds.
• TimeDiff: TimeDiff was a significant positive predictor of APS. This suggests that participants with larger time-estimation errors (i.e., lower accuracy) prefer faster visual motion.
• VDS-J: VDS-J scores negatively predicted APS. Higher visual discomfort (VDS-J) predicted lower APS (i.e., slower preferred speeds).

**Table 4.** Multiple linear regression results for predicting Average Preferred Speed.

Predictor	B	SE B	β	t	p	VIF	95% CI for B
(Constant)	51.49	58.13		0.886	0.380		
STAI	9.54	2.76	0.44	3.46	0.001	1.128	3.92 to 15.17
TimeDiff	4.77	1.69	0.36	2.83	0.007	1.094	1.38 to 8.16
VDS-J	-4.06	1.68	-0.33	-2.42	0.019	1.199	-7.42 to -0.70

*Note:* N = 50. *B*, unstandardized regression coefficient; *SE B*, standard error of the coefficient; *β* = standardized coefficient; VIF, Variance Inflation Factor; STAI, State-Trait Anxiety Inventory; TimeDiff, Absolute Difference between Estimated Time and Actual Time; VDS-J, Visual Discomfort Scale Japanese version scores.

The standardized coefficients ( *β*) indicated that STAI scores ( *β* = 0.44) had the strongest relative influence on APS, followed by TimeDiff ( *β* = 0.36) and VDS-J scores ( *β* = -0.33).

The variance inflation factor (VIF) values for all predictors were close to 1, indicating no serious multicollinearity issues in the final model. Other variables, including HRDiff, PHR, AHR, ST, and TQ-J scores, did not contribute significantly to the prediction of APS in this model.

These findings provide insight into factors influencing preferred RDM speed. However, it is important to note that while the model explains a substantial portion of the variance in the APS (31.8%), unexplained variability remains, indicating that other factors not included in this model may also influence the preferred bubble tube speed.

## Discussion

This study aimed to investigate the relationship between various physiological and psychological factors and APS during RDM stimulation. Using Spearman’s correlation and multiple regression analyses, we identified several significant predictors of APS, providing insight into the interplay between interoceptive sensitivity, subjective time perception, visual discomfort levels, anxiety levels, and the preferred speeds for bubble tubes.

Our pattern of associations aligns with adjacent literatures on sensory processing, neurodivergent experience, and multi-sensory therapeutic environments. Sensory-integration frameworks propose individual differences in sensory thresholds and modulation that shape comfort under dynamic input ( Dunn, 2001; Lane et al., 2019). In autism research, atypical motion processing and heightened visual sensitivity can bias preferences toward slower, steadier dynamics when discomfort is high ( Robertson & Baron-Cohen, 2017). Clinical and community studies of multi-sensory environments further indicate that low-demand, user-controlled inputs reduce distress and improve engagement ( Champagne & Stromberg, 2004; Scanlan & Novak, 2015; Unwin et al., 2022, 2024). Framing bubble-tube speed as a design-tunable parameter therefore accords with theory and practice, while our data identify person-level predictors that can guide initial settings and individualized adjustments.

The significant positive correlation between the absolute difference between perceived and actual heart rate (HRDiff) and APS ( *rs* = 0.49, 95% *CI* [.238, .681], *p* < .001) underscores the pivotal role of interoceptive sensitivity in shaping the preferred RDM stimulation speeds. Interoceptive sensitivity and sensory processing are related via a clear mechanistic pathway. Enhanced interoceptive sensitivity enables more precise detection of internal physiological states, which, in turn, facilitates higher temporal resolution in sensory processing. This heightened internal awareness allows individuals to detect subtle physiological responses to sensory stimuli more accurately. Recent research by Grist et al. (2023) supports this connection by demonstrating that interoceptive awareness is correlated with sensory processing capabilities in neurotypical children, suggesting this relationship is fundamental to human development.

People with higher interoceptive sensitivity preferred slower visual motion, which may reflect two complementary mechanisms. First, reduced speed decreases information load and prevents sensory system overload. Second, slower presentation speeds provide extended processing windows, allowing for more thorough signal integration. This pathway enhances sensory comfort in two ways: by reducing sensory overload, and by improving processing precision. Slower information presentation lowers neural stress and fatigue, while better signal-to-noise ratios and prediction accuracy promote psychological ease. Together, these factors enable effortless engagement with the sensory environment, facilitating effective tracking and integration of stimuli for an optimal sensory experience.

The positive correlation between anxiety levels and APS ( *rs* = 0.31, 95% *CI* [.076, .582] *p* = .028), as confirmed by the multiple regression analysis ( *β* = 0.44, *p* = .001), suggests that faster RDM stimulation speeds may be preferred by individuals with higher anxiety levels. This finding is consistent with that of Park and Youn (2022) who found that high-intensity visual stimuli can replenish cognitive resources and reduce anxiety. Faster speeds may induce a state of physiological arousal that counteracts the heightened arousal associated with anxiety, thereby promoting relaxation and calmness.

The multiple regression analysis not only confirmed the independent contribution of anxiety levels to the APS, but also revealed the significant influence of subjective time perception. Although not significantly correlated with APS in the correlation analysis, the Absolute Difference between Estimated Time and Actual Time (TimeDiff) emerged as a predictor in the regression model ( *β* = 0.36, *p* = .007), suggesting that subjective time perception may interact with other factors to influence the preferred RDM stimulation speed in a complex manner. Individuals with lower subjective time-perception accuracy, in the same way as those with lower interoceptive sensitivity, may perceive faster RDM stimulation speeds as more comfortable. This finding suggests a potential link between time perception and interoceptive processing, as proposed by Di Lernia et al. (2018). Subjective time appears to covary with interoceptive processes: people with greater interoceptive accuracy may prefer slower visual motion, consistent with our observation that larger time-estimation errors were associated with faster preferred speeds. Furthermore, while visual discomfort did not show significant correlations with APS in the correlation analysis, the multiple regression analysis revealed additional effects. Specifically, higher VDS-J scores were significantly associated with lower APS ( *β* = -0.33, *p* = .019). These results indicate that although visual discomfort may not independently predict the APS, it contributes to the overall model of the preferred speed. This suggests that individuals with higher visual discomfort levels perceive slower speeds as more comfortable. This finding aligns with that of Penacchio et al. (2023) who suggested that slower visual stimulus speeds can alleviate discomfort in individuals with visual sensitivity. This emphasizes the importance of tailoring the sensory stimuli to individual comfort levels to maximize the therapeutic benefits of RDM stimulation.

These insights contribute to our understanding how physiological and psychological factors relate to preferred RDM speed and suggest potential pathways for designing sensory interventions. Our findings showed that the participants’ preferred RDM stimulation speeds varied according to their physiological and psychological factors, underscoring the need for personalized design in bubble tubes and sensory rooms.

### Limitations

This study has several limitations that qualify the interpretation of the findings. First, the sample size (N = 50) constrains statistical power and external validity. Although the final regression model explained a meaningful proportion of variance, estimates may be unstable in small samples, and the precision of effects is limited. Replication with larger, prospectively powered cohorts is needed to refine effect sizes and improve generalizability.

Second, the multiple linear regression analysis used a stepwise variable-selection procedure after considering several candidate predictors. Data-driven selection can capitalize on chance and inflate Type I error, even when diagnostics such as VIF are satisfactory. Future work should confirm the model using a priori–specified predictors, cross-validation or a hold-out set, and consider regularized regression (e.g., ridge/LASSO) to reduce overfitting.

Third, the sample comprised a non-clinical convenience group. As such, the results may not generalize to autistic or other neurodivergent populations, nor to broader clinical settings. Multi-site studies that include autistic participants and other relevant user groups are required to establish transportability.

Fourth, several measures relied on self-report questionnaires (VDS-J, TQ-J, STAI), which are susceptible to response styles and situational influences. Moreover, time perception was indexed by a single 60-s estimation task, and interoception by the heartbeat-tracking task; both are single-method operationalizations with known variability. Future work should adopt multi-method batteries (e.g., heartbeat discrimination or respiratory interoception tasks; MAIA for sensibility) and report internal consistency/test–retest where applicable.

Fifth, regarding ecological validity, the upward random dot motion (RDM) stimulus isolates motion speed but does not reproduce the full chromatic, acoustic, and tactile dynamics of physical bubble tubes. Consequently, the preferred speeds identified here should be treated as laboratory proxies. Follow-up studies should verify whether preferences translate to device-controlled bubble tubes or video stimuli and to multi-sensory environments with user-controlled settings.

Finally, the study was exploratory and was not preregistered. While we mitigate this by sharing de-identified data, SPSS outputs, and a step-by-step analysis recipe, future research would benefit from preregistration, independent replication, and expanded physiological recording (e.g., HRV/EDA during stimulation) to test mechanistic accounts.

## Conclusions

This study provides insight into the interplay between physiological and psychological factors that influence preferred RDM stimulation speeds. Our findings reveal that interoceptive sensitivity, subjective time perception, visual discomfort, and anxiety are significant predictors.

These results demonstrate that individuals with lower interoceptive sensitivity tend to prefer faster RDM stimulation speeds, possibly as a compensatory mechanism for reduced internal bodily awareness. Observed associations between interoceptive sensitivity and preferred stimulation speed highlight the value of personalizable sensory environments—e.g., adjustable bubble-tube speeds—that account for individual physiological differences.

Anxiety levels emerged as a significant factor, with anxiety significantly and positively correlated with preferred RDM speed. This finding suggests that faster and more intense visual stimuli may have applications in anxiety management. Saiu and Grosso (2022) demonstrated that combined audio-visual stimulation significantly reduced anxiety levels, systolic blood pressure, and heart rate in patients undergoing surgery, supporting the potential of multi-sensory interventions in anxiety management.

Subjective time-perception ability was also related to the preferred RDM speed, with participants showing lower accuracy tending to prefer faster speeds. Previous research has shown that interoceptive focus significantly influences subjective time perception, with heightened interoceptive awareness amplifying the time-dilating effects of fear and the time-accelerating effects of amusement ( Pollatos et al., 2014). This, in turn, may have affected participants’ perceptions of comfort in response to RDM stimulation speeds.

Although visual discomfort was not independently correlated with preferred speed in the Spearman correlation analysis, it emerged as a significant predictor in the multiple regression analysis. Specifically, participants with higher visual sensitivity tended to prefer slower speeds. This finding highlights the importance of designers considering individual levels of visual discomfort when designing visual stimuli in bubble tubes or sensory rooms and offering tailored sensory experiences that cater to each person’s diverse sensory needs.

As Ziegler (2015) noted in her research on multi-sensory design in healthcare settings, environments that allow users to customize sensory inputs contribute to stress reduction and improved well-being. Garzotto et al. (2019) developed an extensible multilayer software and hardware platform to connect and manage different devices in a sensory room, thereby enabling therapists to fully customize activities in multi-sensory environments for different children. These findings suggest that personalizable sensory environments—for example, installations with adjustable bubble-tube speeds—may better accommodate diverse sensory needs and support user comfort and self-regulation. Building on our previous research ( Su et al., 2024), which demonstrated that sensory-hypersensitive individuals have greater variations in their preferences for wallpaper colors and patterns, this study further underscores the necessity for customizable sensory rooms tailored to each person’s unique sensory needs.

These findings should be interpreted in light of the study’s limitations. A key limitation is the lack of clinical characterization: participants were not screened for neurodevelopmental or anxiety disorders, and medication status was not recorded. Consequently, generalizability to autistic or clinically anxious populations is uncertain.

In conclusion, this study enhances our understanding of the factors influencing preferred bubble tube speeds and lays the groundwork for the development of more personalized and effective sensory interventions to improve outcomes for individuals with diverse sensory needs.

### Ethical considerations

This study recruited 50 adult participants (27 females) aged 22–35 years (M = 25.94, SD = 2.74) from the University of Tsukuba between December 25, 2023, and March 22, 2024. The research was conducted in accordance with the principles of the Declaration of Helsinki and was approved by the Institutional Review Board (IRB) of the Institute of Art and Design, the University of Tsukuba (IRB No. [GEI021-15]) on March 22, 2022. Prior to participation, all participants provided written informed consent. To ensure privacy, all participant data were anonymized, and no identifying information was collected or retained.

Written informed consent for publication of the participants details was obtained from the participants.

## Data Availability

All data, materials, and software are available under the Creative Commons Attribution 4.0 International (CC-BY 4.0) license. Zenodo: Preferred Bubble Tube Speed and Physiological–Psychological Factors. DOI: 10.5281/zenodo.14633771 ( Su, A., 2025b). De-identified participant-level dataset (N = 50) containing trial-level and summary variables used in the analyses. Zenodo: Preferred Bubble Tube Speed & Physiological and Psychological Factors — SPSS Outputs and Analysis Recipe (v1.0). DOI: 10.5281/zenodo.17045820 ( Su, A., 2025c). Zenodo: Checklist for “Exploring the relationship between preferred bubble tube speeds in sensory rooms and physiological–psychological factors: A study on interoceptive sensitivity, subjective time perception, visual discomfort levels, and anxiety levels”. DOI: 10.5281/zenodo.14633918 ( Su, A., 2025a). License: CC-BY 4.0. Zenodo: Scales used to assess participants’ levels of visual discomfort and anxiety. DOI: https://doi.org/10.5281/zenodo.14770842 ( Su, A., 2025f). GitHub: https://github.com/Lem0n-SAJ/RDM_Program_Su Zenodo (source code): https://doi.org/10.5281/zenodo.14795461 ( Su, A, 2025d) Zenodo (Windows executable): https://doi.org/10.5281/zenodo.14795194 ( Su, A, 2025e) License: MIT Data are available under the terms of the Creative Commons Attribution 4.0 International license (CC-BY 4.0).
