# Supplementary material for: Exploring the relationship between preferred bubble tube speeds in sensory rooms and physiological–psychological factors: A study on interoceptive sensitivity, subjective time perception, visual discomfort levels, and anxiety levels
Source: F1000Res. 2026 Mar 27;14:214. Originally published 2025 Feb 14. [Version 2] doi: 10.12688/f1000research.161090.2 (PMC13263667; doi:10.12688/f1000research.161090.2)
Supplement: Peer review history

# Reviewer report: Reviewer response for version 2

**DOI:** 10.5256/f1000research.195119.r489070

**Published:** 2026-06-26

**Authors:** Mrinmoy Chakrabarty

**Affiliations:** 1 Indraprastha Institute of Information Technology Delhi, New Delhi, Delhi, India

Summary: 

 The study addresses a practically relevant question: whether preferred bubble-tube motion speed relates to interoception, time perception, visual discomfort, and anxiety. The use of an RDM paradigm to isolate motion speed and the open-science practices are its strength. The revised manuscript is improved, but the following issues require further attention. 

 Major comments: 

 1) The manuscript frames its clinical applications around autism, sensory hypersensitivity, and anxiety. However, the study actually used a non-clinical convenience sample of college students. While I appreciate that the authors added this limitation to the text, the Discussion and Conclusion sections still need to be toned down. Any claims about anxiety management or sensory-room design for clinical or autistic populations must be rephrased as ideas for future research rather than proven facts.

 2) I appreciate the improvements made to the predictor descriptions and the stepwise regression limitations. Because of the relatively modest sample size, these findings are best interpreted as exploratory. The manuscript would benefit from either including a full model in the supplements or softening the conclusions regarding predictor importance. Finally, please expand slightly on the variables that became significant in the regression despite weak bivariate links, as this likely points to interesting suppression effects or shared variance.

 3) I appreciate that the authors explicitly noted the lack of multiplicity correction and provided their rationale. However, because the paper still interprets several significant correlations, these findings should be treated with caution. To address this, I suggest either running a quick FDR-based sensitivity analysis or using suitable language throughout the Results and Discussion to clarify that these findings are strictly exploratory and descriptive.

 4) To help readers fully understand the methodology, it would be helpful to clarify a few details regarding the procedure and task setup. Please specify the exact order of the tasks and questionnaires, noting whether the STAI, VDS, and TQ were administered before or after the RDM task. Additionally, it would be helpful to know if participants were given breaks (numbers, approx. durations) during the session. Finally, please ensure the key RDM implementation details relevant to motion perception, such as dot lifetime and the dot replacement rule are clearly detailed if they aren't already mentioned elsewhere.

 Minor comments: 

 1) To ensure a smooth flow of the Introduction, the authors may consider adding a short paragraph that explicitly links the focal constructs together.

 2) Please ensure that causal/mechanistic language is consistently toned down where the data are correlational.

Is the work clearly and accurately presented and does it cite the current literature?

Partly

If applicable, is the statistical analysis and its interpretation appropriate?

Partly

Are all the source data underlying the results available to ensure full reproducibility?

Yes

Is the study design appropriate and is the work technically sound?

Yes

Are the conclusions drawn adequately supported by the results?

Partly

Are sufficient details of methods and analysis provided to allow replication by others?

Yes

Reviewer Expertise:

Cognitive Neuroscience

I confirm that I have read this submission and believe that I have an appropriate level of expertise to confirm that it is of an acceptable scientific standard, however I have significant reservations, as outlined above.

# Reviewer report: Reviewer response for version 2

**DOI:** 10.5256/f1000research.195119.r474368

**Published:** 2026-06-12

**Authors:** Sarah M Haigh

**Affiliations:** 1 University of Nevada Reno, Reno, Nevada, USA

I can see that revisions have already been made in response to comments and so I will endeavor not to add contradiction.

 The topic is interesting and highly relevant based on the current conversation regarding understanding sensory sensitivity in clinical and nonclinical populations and the use of sensory rooms for those who need them. However, I have a couple of questions and suggestions for clarifying the study.

 The introduction is focused on clinical (particularly autistic) populations and yet the sample recruited were non-clinical and no subclinical measures related to autism or other conditions were collected, except for anxiety. This in itself would be fine, but there were no checks to see if any of the participants had a diagnosis, which throws into question whether the sample were non-clinical and if there were any medication confounds. Is there any way the authors can address this concern? I see it is included towards the end of the discussion but does not resolve the use of these data when understanding the variability in preferred bubble speed.

 The introduction introduces multiple components and currently reads a little choppy. Subheadings or a light introduction into the topics to be covered might help readability (minor concern). May also help to highlight the variability in some of the scales (anxiety, visual discomfort etc.) in the general population that tends to be more extreme in clinical populations, making the non-clinical/convenience sample for this study seem more appropriate. In other words, taking a course look at variability in preferred speed in relation to a variety of scales without subdividing would be representation of the typical populations walking through an airport (for example) and may want to use a sensory room.

 For methods, please clarify which measures were recorded once and why. Are there concerns about task uncertainty affecting results? The counting tasks do appear to be a bit unusual. Why these over more standard temporal precision tasks?

 Highlighting what the direction of the DVs mean consistently would help. For example, counting 60 seconds was calculated as absolute difference with actual, why? Negative values would also be interesting. Closer to zero means more accurate, correct? Same for the heartbeat counting task, absolute measures only?

 Why trypophobia? Comes out of nowhere and there is a debate as to the relationship with visual discomfort. Can the authors add a description of the questionnaires to give the reader a better understanding of what is being asked? Some measures, for example of visual discomfort, only ask about one component (reading or light sensitivity) whereas others are more comprehensive. This ensures that the reader does not have to read the citations to understand what is going on here.

 Seem to be multiple comparisons being conducted - any correction for Type 1 error? Regression analysis only reports significant effects but more variables were included in the model? Unclear as to how the limited factors were decided upon. 

 Scatterplots showing the data would be helpful to illustrate relationships. Were measures related to one another?

 Minor point in discussion: Penacchio et al. (2023) reference is stated to say something about motion and visual discomfort. As far as I am aware, it does not. Can the authors clarify what they are referring to?

Is the work clearly and accurately presented and does it cite the current literature?

Partly

If applicable, is the statistical analysis and its interpretation appropriate?

Partly

Are all the source data underlying the results available to ensure full reproducibility?

Yes

Is the study design appropriate and is the work technically sound?

Yes

Are the conclusions drawn adequately supported by the results?

Partly

Are sufficient details of methods and analysis provided to allow replication by others?

Partly

Reviewer Expertise:

Visual discomfort

I confirm that I have read this submission and believe that I have an appropriate level of expertise to confirm that it is of an acceptable scientific standard, however I have significant reservations, as outlined above.

# Reviewer report: Reviewer response for version 1

**DOI:** 10.5256/f1000research.177078.r375957

**Published:** 2025-08-22

**Authors:** Sarune Savickaite

**Affiliations:** 1 University of Exeter, Exeter, UK

This study explored how individuals’ physiological and psychological characteristics affect their preferred speeds of bubble tube stimulation in sensory rooms, using a simulated upward-moving visual task. Results showed that people with higher interoceptive sensitivity preferred slower speeds, while those with higher anxiety or less accurate time perception preferred faster speeds. Visual discomfort also played a role, with those experiencing more discomfort tending to prefer slower visual motion. A combination of interoceptive accuracy, anxiety levels, time estimation ability, and visual sensitivity significantly predicted preferred stimulation speed. The findings highlight the importance of personalized sensory environments tailored to individual needs for optimal therapeutic impact.

 1. Terminology.  Use “autism” instead of “ASD” throughout for a more inclusive and widely accepted terminology, especially in line with neurodiversity research.

 2. Avoid Ableist Language. Refrain from using medicalized or deficit-based terms such as “impairment.” Instead, adopt terminology grounded in the social model of disability, which focuses on societal barriers rather than individual deficits.

 3. Identity-First Language. Use “autistic individuals” rather than “individuals with autism” to align with current preferences within the autistic community and neurodiversity literature.

 4. Improve Rationale Structure. Streamline the background by clearly introducing the key constructs (e.g., interoception, time perception, anxiety, visual discomfort), then presenting a focused rationale for the current study. Avoid repeating the aim multiple times.

 5. Reference Gaps. In the paragraph starting “However, the precise mechanisms by which the bubble tubes induce relaxation remain poorly understood…”, add references to prior sensory integration or multi sensory environments research to contextualize the claim.

 6. Existing Research. Although this is a novel area, relevant adjacent literature does exist. Draw clearer parallels to studies in sensory processing, neurodivergent experience, and multi-sensory therapeutic environments.

 7. Conceptual Clarity

 Interoception. Define which aspects of interoception are relevant (e.g., accuracy, awareness, sensibility), and clarify why they matter for this context. Reference foundational literature (e.g., Garfinkel et al., 2015; Critchley & Garfinkel, 2017).

 Time Perception. Explicitly explain how time perception relates to interoception and anxiety in this study. Currently, it appears disconnected.

 Anxiety and Visual Discomfort. Define anxiety more clinically (e.g., state vs. trait), and expand discussion of visual discomfort with more references. Both constructs are complex and under-contextualized in the paper.

 8. Participant Characteristics. Clarify whether participants were asked about clinical diagnoses (e.g., anxiety, neurodevelopmental conditions), given their relevance to the research aims. If not assessed, provide a justification.

 9. Stimuli Choice. Justify the use of Random Dot Motion (RDM) stimuli, particularly in the context of interoception and neurodiversity research, where its utility is not well-established.

 10. Measures.  Provide a clearer, consolidated presentation of the scales and tasks used to measure anxiety, interoception, time perception, and visual discomfort—ideally in a dedicated table with psychometric properties and validation references.

 11. Open Science Considerations. While SPSS is a common statistical tool, open science practices would benefit from clear data and protocol sharing. Explicitly state where readers can access the materials.

 12. Statistical Rationale. Explain why Spearman’s correlation was used (e.g., non-normal distribution), and clarify whether regression models were corrected for multiple comparisons. Justify the model selection process more clearly.

 13. Sample Size Concerns. The relatively small sample (N=50) may limit the power and generalizability of the regression analysis. Discuss limitations related to sample size and possible risks of overfitting or underpowered findings.

 14. Streamline Results. Reporting of statistics (e.g., means, SDs, p-values, CI) could be streamlined for clarity. Use visual aids (tables or figures) to consolidate key outcomes for easier interpretation.

 15. Limitations Section: Explicitly address the study’s limitations, including sample size, generalizability, reliance on self-report measures, and the exploratory nature of the design. This is currently underdeveloped and needed for transparency.

Is the work clearly and accurately presented and does it cite the current literature?

Partly

If applicable, is the statistical analysis and its interpretation appropriate?

Partly

Are all the source data underlying the results available to ensure full reproducibility?

No

Is the study design appropriate and is the work technically sound?

Partly

Are the conclusions drawn adequately supported by the results?

Partly

Are sufficient details of methods and analysis provided to allow replication by others?

Partly

Reviewer Expertise:

I work with neurodiversity, perceptual differences and interoception. This is my area of expertise and this article requires major revisions.

I confirm that I have read this submission and believe that I have an appropriate level of expertise to confirm that it is of an acceptable scientific standard, however I have significant reservations, as outlined above.

## References

[1] : Discrepancies between dimensions of interoception in autism: Implications for emotion and anxiety. *Biological Psychology* .2016;114: 10.1016/j.biopsycho.2015.12.003 117-126 10.1016/j.biopsycho.2015.12.003 26724504

[2] : Interoception and emotion. *Current Opinion in Psychology* .2017;17: 10.1016/j.copsyc.2017.04.020 7-14 10.1016/j.copsyc.2017.04.020 28950976

# Response

**Published:** 2026-01-14

**Authors:** Shinichi Koyama

**Affiliations:** Institute of Art and Design, University of Tsukuba, Tsukuba, Ibaraki Prefecture, Japan

Response to reviewer 2.

 Thank you very much for your valuable comments.

 1. Terminology.  Use “autism” instead of “ASD” throughout for a more inclusive and widely accepted terminology, especially in line with neurodiversity research.

**=>** We replaced ASD with autism throughout the paper. We also replaced autism disorder-based terminology and sensory processing with neurodiversity-affirming language.

 2. Avoid Ableist Language. Refrain from using medicalized or deficit-based terms such as “impairment.” Instead, adopt terminology grounded in the social model of disability, which focuses on societal barriers rather than individual deficits.

**=>** We removed medicalized/deficit-based phrasing and reframed descriptions using the social model of disability.

    address these challenges => provide sensory support (Page 2, Line 48)

    functional abilities => agency within everyday environments (Page 2, Line 50)

    history of aggression => experience heightened distress responses (Page 3, Line 67)

 3. Identity-First Language. Use “autistic individuals” rather than “individuals with autism” to align with current preferences within the autistic community and neurodiversity literature.

**=>** We now use autistic children/adults/people/populations/participants rather than individuals with autism, except when citing instruments or studies that use diagnostic labels (Pages 3, 5, 7, 13).

 4. Improve Rationale Structure. Streamline the background by clearly introducing the key constructs (e.g., interoception, time perception, anxiety, visual discomfort), then presenting a focused rationale for the current study. Avoid repeating the aim multiple times.

**=> **We revised the Introduction to follow a four-step structure: background, mechanisms/needs, key constructs (interoception, time perception, anxiety, and visual discomfort), and presentation of the present study and hypotheses. We streamlined the rationale and removed duplicated statements of the aim to ensure a clearer progression (Pages 2-5).

 5. Reference Gaps. In the paragraph starting “However, the precise mechanisms by which the bubble tubes induce relaxation remain poorly understood…”, add references to prior sensory integration or multi-sensory environments research to contextualize the claim.

**=>** We added key citations (Champagne & Stromberg, 2004; Sutton et al., 2013; Scanlan & Novak, 2015) (Page 3, Line 83) to contextualize multisensory modulation mechanisms.

 6. Existing Research. Although this is a novel area, relevant adjacent literature does exist. Draw clearer parallels to studies in sensory processing, neurodivergent experience, and multi-sensory therapeutic environments.

**=> **We strengthened the parallels to existing research in sensory processing, neurodivergent experiences, and multi-sensory environments. Specifically, we incorporated evidence from observational studies and systematic reviews (e.g., Breslin et al., 2020, Page 3, Lines 86-89; Haig & Hallett, 2023, Page 3, Lines 67-71) to contextualize the use of sensory equipment and highlight the variable outcomes across different populations. These additions underscore the necessity of identifying person-level moderators, such as motion processing differences in autistic individuals, to better understand engagement in therapeutic spaces.

 7. Conceptual Clarity

 Interoception. Define which aspects of interoception are relevant (e.g., accuracy, awareness, sensibility), and clarify why they matter for this context. Reference foundational literature (e.g., Garfinkel et al., 2015; Critchley & Garfinkel, 2017). Time Perception. Explicitly explain how time perception relates to interoception and anxiety in this study. Currently, it appears disconnected.

 Anxiety and Visual Discomfort. Define anxiety more clinically (e.g., state vs. trait), and expand discussion of visual discomfort with more references. Both constructs are complex and under-contextualized in the paper.

**=> **We expanded the conceptual definitions of interoception in the Introduction (Page 4, Line 90). We justified our specific focus on interoceptive accuracy as an objective index (Page 4, Line 94).

**=>** Beyond interoception, we also strengthened the theoretical framework by:

 For interoception, we now explicitly define accuracy, sensibility, and awareness, citing foundational literature (Garfinkel et al., 2015; Critchley & Garfinkel, 2017). Subjective Time Perception: linking it to interoception and anxiety (Page 4). Visual Discomfort: Elaborating on the mechanisms of pattern-glare and the impact of spatial/temporal statistics (Page 4). Anxiety: Clarifying the distinction between state and trait anxiety as measured by the STAI short form (Pages 4-5).

 8. Participant Characteristics. Clarify whether participants were asked about clinical diagnoses (e.g., anxiety, neurodevelopmental conditions), given their relevance to the research aims. If not assessed, provide a justification.

**=> **We clarified that clinical diagnoses (such as anxiety or neurodevelopmental conditions) were not recorded, as the study was designed as a foundational step to investigate person-level predictors of sensory comfort within a general population sample. To address this, we justified this non-clinical focus in the Methods section (Page 6, Line 171) and added a corresponding acknowledgment in the Limitations section (Page 13, Line 419). In the latter, we further propose that future follow-up work should specifically involve autistic and other neurodivergent cohorts to validate these findings and enhance the generalizability of the results within clinical settings.

 9. Stimuli Choice. Justify the use of Random Dot Motion (RDM) stimuli, particularly in the context of interoception and neurodiversity research, where its utility is not well-established.

**=>** We added a dedicated rationale for the use of RDM stimuli, emphasizing their utility in isolating the motion-speed component of bubble-tube viewing while minimizing confounding variables such as color, texture, and form. In the revised Introduction (Page 5, Line 148), we explicitly linked the RDM paradigm to canonical neurophysiological work in the MT/V5 area and its established application in neurodiversity research, particularly in assessing global motion processing in autistic populations. Furthermore, we have expanded the Methods section to clarify how the RDM provides precise parametric control over the upward motion speed while maintaining a constant dot density and luminance. These additions justify the translational value of RDM as a proxy for bubble-tube motion within the context of interoceptive and sensory-processing research.

 10. Measures.  Provide a clearer, consolidated presentation of the scales and tasks used to measure anxiety, interoception, time perception, and visual discomfort—ideally in a dedicated table with psychometric properties and validation references.

**=> **In response to the request for a more consolidated presentation of our measures, we reorganized all experimental tasks and scales into two comprehensive tables (Page 23). Table 1A summarizes the experimental tasks (e.g., interoception and time perception), whereas Table 1B details the questionnaires used for anxiety and visual discomfort. These tables provide a clear overview of the key settings, scoring procedures, psychometric properties, and validation references with consistent citations. These changes significantly improve the transparency of our operational definitions and streamline the referencing of specific constructs in the Results section.

 11. Open Science Considerations. While SPSS is a common statistical tool, open science practices would benefit from clear data and protocol sharing. Explicitly state where readers can access the materials.

**=> **We fully agree with the importance of open science practices and included a dedicated Data Availability Statement (Page 16, Line 510) with persistent DOIs to ensure the transparency and reproducibility of our findings. We provide a comprehensive suite of materials, including de-identified participant-level data in .sav format, original SPSS output files (.spv), and a step-by-step analysis protocol in PDF format. As noted in the revised Methods section (Page 9, Line 269), all statistical procedures were conducted via the graphical interface of IBM SPSS Statistics v27. By sharing the original output files along with a detailed "analysis recipe," we enable other researchers to fully verify and replicate our analytical steps.

 12. Statistical Rationale. Explain why Spearman’s correlation was used (e.g., non-normal distribution), and clarify whether regression models were corrected for multiple comparisons. Justify the model selection process more clearly.

**=> **Response: We updated the Methods and Results sections to provide a more detailed statistical rationale. First, we clarified that Spearman’s rank correlation was employed because several variables exhibited non-normal distributions and outliers, as determined by skewness and kurtosis (Page 8, Lines 256-263). Regarding model selection, we now transparently describe the stepwise regression criteria (entry p < .05, removal p > .10) while adding caution regarding potential overfitting (Page 10, Line 314). To address the issue of multiple comparisons, we framed our correlation analyses as exploratory and descriptive, and prioritized the interpretation of effect sizes and two-tailed 95% confidence intervals. We have added a corresponding note in the Limitations section to ensure transparency regarding the risk of Type I errors (Page 13, Lines 424-428).

 13. Sample Size Concerns. The relatively small sample (N=50) may limit the power and generalizability of the regression analysis. Discuss limitations related to sample size and possible risks of overfitting or underpowered findings.

**=>** We acknowledge the limitations associated with our sample size (N = 50) and added a dedicated subsection to the Limitations (Page 13, Lines 419-423) to address the concerns regarding statistical power and generalizability. In this section, we discuss how a relatively small sample size may limit the precision of our estimates and increase the risk of overfitting, particularly in the context of stepwise regression selection.

 14. Streamline Results. Reporting of statistics (e.g., means, SDs, p-values, CI) could be streamlined for clarity. Use visual aids (tables or figures) to consolidate key outcomes for easier interpretation.

**=>** We streamlined the Results section to enhance clarity and interpretability by reducing the statistical density of the text and consolidating key data into tables. Descriptive statistics for all variables are shown in Table 2 (Page 23), allowing for a more focused narrative flow in the text. Throughout the Results section, we now report a standardized and consistent set of statistics, including means, standard deviations, Spearman correlation coefficients (rs) with 95% confidence intervals and p-values, as well as comprehensive regression parameters (B, SE, β, 95% CI, and VIF). This reorganization ensures that all critical outcomes are presented transparently and are easily accessible to the reader while maintaining a concise and readable manuscript.

 15. Limitations Section: Explicitly address the study’s limitations, including sample size, generalizability, reliance on self-report measures, and the exploratory nature of the design. This is currently underdeveloped and needed for transparency.

**=>** We expanded the Discussion section by adding a dedicated Limitations subsection (Page 13) to ensure full transparency and provide a balanced interpretation of our findings. This section comprehensively addresses the statistical constraints of our sample size (N = 50), including precision limits and potential overfitting risks associated with our model selection. We also discuss the generalizability of our results, noting that the use of a non-clinical convenience sample may not fully represent the experiences of autistic or other neurodivergent groups. Furthermore, we acknowledge the reliance on self-report measures and ecological validity considerations when using RDM stimuli as a proxy for physical bubble tubes. Finally, we clarify the exploratory nature of the design and the absence of registration, highlighting how our commitment to sharing open materials mitigates these concerns and supports future replication efforts.

# Reviewer report: Reviewer response for version 1

**DOI:** 10.5256/f1000research.177078.r382825

**Published:** 2025-06-13

**Authors:** Teresa Tavassoli

**Affiliations:** 1 University of Reading, Reading, England, UK

This paper is about an interesting topic of the speed of bubbles and how its relates to interoception and visual discomfort. Here are a few comments :

 In the introduction also mention the work on sensory hyperreactivity and anxiety such as Green et al., 2010 and 2012, MacLennan at el, 2020, 2021, Verhulst et al., 2022. 

 Please comment on why healthy adults were used for this study. The  introduction covers anxiety, autism and mental health conditions and thus it might have been more informative to use one of these groups rather then healthy controls.

Is the work clearly and accurately presented and does it cite the current literature?

Partly

If applicable, is the statistical analysis and its interpretation appropriate?

Yes

Are all the source data underlying the results available to ensure full reproducibility?

Yes

Is the study design appropriate and is the work technically sound?

Yes

Are the conclusions drawn adequately supported by the results?

Yes

Are sufficient details of methods and analysis provided to allow replication by others?

Yes

Reviewer Expertise:

autism, sensory reactivity

I confirm that I have read this submission and believe that I have an appropriate level of expertise to confirm that it is of an acceptable scientific standard.

# Response

**Published:** 2026-01-14

**Authors:** Shinichi Koyama

**Affiliations:** Institute of Art and Design, University of Tsukuba, Tsukuba, Ibaraki Prefecture, Japan

Response to reviewer 1

 Thank you very much for your comment.

 In the introduction also mention the work on sensory hyperreactivity and anxiety such as Green et al., 2012, MacLennan et al, 2020, 2021, Verhulst et al., 2022. Please comment on why healthy adults were used for this study. The introduction covers anxiety, autism and mental health conditions and thus it might have been more informative to use one of these groups rather than healthy controls.

**=>** We have now incorporated the references you mentioned in the Introduction section to better contextualize our work within the existing literature on sensory hyperreactivity and anxiety: Green et al., 2012 (Page 3, Line 55); MacLennan et al., 2020, 2021 (Page 3, Lines 55-56); Verhulst et al., 2022 (Page 3, Line 55-56).

**=>**The purpose of the study was to examine the correlation between sensory sensitivity (ranging from very low to very high sensitivity) and preferred dot speed. We therefore recruited participants with a wide variance in sensitivity, from below average to highly sensitive, regardless of the presence or absence of a clinical diagnosis.
